# An immune response characterizes early Alzheimer’s disease pathology and subjective cognitive impairment in hydrocephalus biopsies

**DOI:** 10.1038/s41467-021-25902-y

**Published:** 2021-09-27

**Authors:** Wenrui Huang, Anne Marie Bartosch, Harrison Xiao, Suvrajit Maji, Elliot H. H. Youth, Xena Flowers, Sandra Leskinen, Zeljko Tomljanovic, Gail Iodice, Deborah Boyett, Eleonora Spinazzi, Vilas Menon, Robert A. McGovern, Guy M. McKhann, Andrew F. Teich

**Affiliations:** 1 Department of Pathology and Cell Biology, Columbia University, New York, NY USA; 2 Taub Institute for Research on Alzheimer’s Disease and the Aging Brain, Columbia University, New York, NY USA; 3 Department of Neurology, Columbia University, New York, NY USA; 4 Department of Neurosurgery, Columbia University, New York, NY USA; 5 Department of Neurosurgery, University of Minnesota, Minneapolis, MN USA

**Keywords:** Computational biology and bioinformatics, Alzheimer's disease, Neurodegeneration

## Abstract

Early Alzheimer’s disease (AD) pathology can be found in cortical biopsies taken during shunt placement for Normal Pressure Hydrocephalus. This represents an opportunity to study early AD pathology in living patients. Here we report RNA-seq data on 106 cortical biopsies from this patient population. A restricted set of genes correlate with AD pathology in these biopsies, and co-expression network analysis demonstrates an evolution from microglial homeostasis to a disease-associated microglial phenotype in conjunction with increasing AD pathologic burden, along with a subset of additional astrocytic and neuronal genes that accompany these changes. Further analysis demonstrates that these correlations are driven by patients that report mild cognitive symptoms, despite similar levels of biopsy β-amyloid and tau pathology in comparison to patients who report no cognitive symptoms. Taken together, these findings highlight a restricted set of microglial and non-microglial genes that correlate with early AD pathology in the setting of subjective cognitive decline.

## Abstract (web summary)

Specific transcriptional changes in microglia associated with Alzheimer’s disease have been reported. Here, the authors show that transcriptional analysis of human hydrocephalus biopsies identifies changes in immune response genes associated with early AD pathology, including cognitive decline.

## Introduction

Ongoing work molecularly characterizing Alzheimer’s disease (AD) autopsy brain tissue has produced a wealth of information about a wide range of pathophysiologic processes in AD^1–4^. Less work has been done to molecularly characterize AD pathology in surgical biopsy tissue from living patients, which is more difficult to obtain but offers unique advantages for studying AD pathophysiology. For example, multiple groups have studied AD pathology in cortical biopsies from normal pressure hydrocephalus (NPH) patients^5–8^, and insights from this work have furthered our understanding of AD pathophysiology. Chronic hydrocephalus in the aging population can occur for a variety of reasons, although the etiology is often unclear. In the absence of a clear etiology, most of these cases are categorized as idiopathic NPH (iNPH). Placing a ventricular shunt is often effective for symptom relief in the setting of NPH/chronic hydrocephalus^7,9,10^, although which patients will have persistent long-term clinical benefit remains to be determined^11^. At the time of shunt placement, a cortical biopsy is often obtained at the brain entry point to look for possible coexistent brain pathology. Perhaps not surprisingly, cortical biopsies taken from elderly NPH patients at shunt placement have been shown to have a relatively high frequency of β-amyloid plaque pathology, ranging from 42 to 67%^8,12^, perhaps due to the fact that early-stage AD in many cases may actually be causing some of the symptoms attributed to NPH/chronic hydrocephalus. Consistent with this hypothesis, the presence of either (1) severe β-amyloid plaque pathology or (2) a cerebrospinal fluid (CSF) AD signature of high phospho-tau/β-amyloid ratio have both been shown to predict a lack of response to shunting^8,13^. Interestingly, unlike β-amyloid plaque pathology, tau pathology is relatively sparse in NPH cortical biopsies^8^, although some studies have found trace tau pathology at higher levels^14^, which is consistent with the fact that most patients coming to shunt surgery do not show severe cognitive impairment (those patients who are pre-AD are likely to be at a Braak stage with sparse to no neocortical tangles). These findings are in agreement with the average age at biopsy for NPH patients, which in the low to mid 70s^5–7^. This age range is significantly lower than the average age of existing AD autopsy cohorts, which are typically in the mid to upper 80s^1,4^, and is close to the average age of initial clinical presentation for AD (75.5 years)^15^. The hypothesis that NPH patients with AD pathology represent a pre-AD group is further buttressed by a recent longitudinal study of 335 NPH patients showing that NPH patients progress to AD at a higher rate than an aged reference population^6^. This study showed that AD pathology on brain biopsy (β-amyloid and tau) is the single best predictor of progression to AD in comparison with other clinical and radiographic metrics. Nevertheless, there were patients in this study (in accordance with previous published work^16–18^) who have AD pathology and do not progress to AD. It should also be noted that the confounding factor of hydrocephalus is present in any NPH cohort. Thus, all of the above studies of NPH patients as a pre-AD group should be interpreted with these caveats in mind (see “Discussion”).

Recent technological advancements in RNA-sequencing (RNA-seq) have led to the generation of transcriptomic datasets from post-mortem brain tissue of patients affected by AD^1–4^. Analysis of this data has shed light on many aspects of AD pathophysiology, such as highlighting the similarities and differences in microglial gene expression between mouse models of AD and human AD autopsy tissue^19^, as well as defining microglial subtypes that may be relevant in human AD^20^. Surgical biopsies from hydrocephalus patients have already proved useful for understanding the clinical consequences of early AD pathology and may also be useful for understanding early transcriptomic changes in AD. Note that, in addition to the advantages mentioned above, surgical biopsies are also free of gene expression changes that accompany end-of-life hypoxia/apoptotic state, as well as any changes in RNA caused by post-mortem degradation, and all cognitive and clinical data curated from the patients’ peri-operative charts are contemporaneous with the time of tissue acquisition. Thus, surgical biopsies from NPH patients represent a valuable opportunity to examine the transcriptomic profile of brain tissue in living patients with early AD pathology.

In this work, we report RNA-seq data from 106 NPH cortical biopsies and correlate changes in gene expression with co-morbid AD pathology in this patient population. Analysis of these biopsies shows a restricted set of microglial and non-microglial genes that correlate with histological measurements of β-amyloid and tau pathology primarily in patients who report subjective cognitive impairment. Specifically, we identify a gene expression module enriched for murine disease-associated genes that positively correlates with AD pathology and a module enriched for murine homeostatic genes that negatively correlates with AD pathology, and in aggregate, this is more consistent with the existing mouse literature than other publicly available AD autopsy datasets^1,2,4,19,21,22^. Finally, these microglial modules are also correlated with microglial plaque association and changes in microglial morphology, and this change is not sensitive to cognitive status. Taken together, these data suggest that an initial microglial response to AD pathology is associated with accumulating pathology, non-microglial cell responses, and patient-reported cognitive status.

## Results

**Fig. 1:**
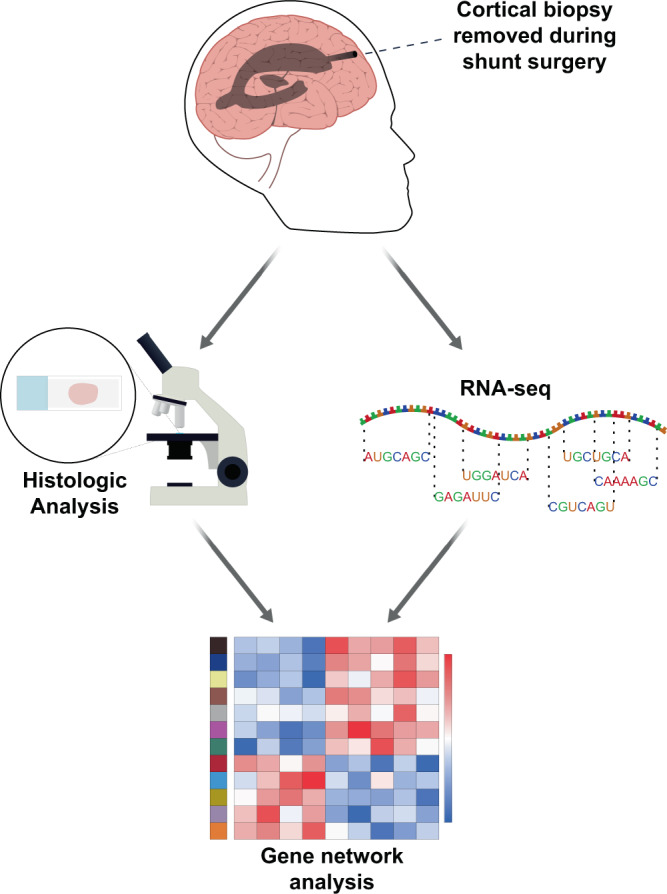
Biopsies removed for ventricular shunting in the operating room are immediately split in half. Half of the biopsy is frozen in liquid nitrogen and sent for RNA-seq. The other half is formalin fixed and paraffin embedded for subsequent pathologic analysis (see “Methods”).

In this study, we examine changes in gene expression that accompany early AD pathology in cortical biopsies that were removed in the course of shunt placement for NPH and compare the results with AD pathology on histology and contemporaneously gathered cognitive data (see Fig. 1 for our workflow and “Methods” for further details on our cohort). Specifically, we performed RNA-seq on 106 biopsies from NPH patients, with an average age of 74.9 years. In all cases, patients were shunted for chronic hydrocephalus by the same surgical team, and biopsies were taken from a specific area of frontal cortex (2/3 of our cohort) or parietal cortex (1/3 of our cohort). The decision to shunt/biopsy in frontal or parietal cortex was made by the surgeon based on cosmetic considerations (see “Methods”). Changes in gene expression that correlate with AD pathology in our samples trend similarly in frontal and parietal cortex (see Supplementary Data 1), and even when we combine all samples, very few individual genes reach statistical threshold (see below).

### Immune/microglia-specific genes are strongly correlated with NPH AD pathology

We initially processed our RNA-seq data by regressing out variability in gene expression not associated with our primary variables of interest (β-amyloid plaque and tau pathology)^23^. β-Amyloid plaques were counted per square millimeter area on slides of tissue immunostained with 6E10 antibody. In order to quantify tau, we devised a rating scale to grade the minimal degree of tau pathology seen in NPH biopsy slides immunostained with AT8 antibody (see Supplementary Fig. 1 for examples of each grade). Grade 0 was given to biopsies with no tau pathology (*n* = 42). Grade 1 was given to biopsies that have any tau pathology at all, usually one or more dystrophic neurites (*n* = 39). Grade 2 was given to biopsies that have at least one tau-positive neuron or neuritic plaque (*n* = 18). Grade 3 was reserved for biopsies with tau pathology evenly distributed throughout the biopsy (*n* = 7).

Initial analysis of the data identified a restricted set of 38 genes that passed false discovery rate (FDR) of 0.1 at the individual gene level that correlated with either β-amyloid and/or tau burden. Indeed, one of the most striking things about our initial analysis is the overall consistency of gene expression signatures in these biopsies, especially given the large-scale changes in gene expression seen in many other autopsy-based cohorts that include brains with clinical AD^1–4^. Note that our sample size of 106 is less than several well-known autopsy cohorts^1,4^. While sample size could contribute to the lower number of significant genes in this study, it should also be noted that the correlations in this study are calculated somewhat differently from correlations reported in previous studies of AD autopsy tissue^1,4^. Specifically, we are correlating changes in gene expression to AD pathology quantified in an immediately adjacent piece of tissue. In contrast, other datasets usually correlate gene expression in a single piece of tissue with global metrics of AD pathology or with AD pathology in tissue that is not necessarily contiguous with the tissue used for RNA-seq, all of which might lead to weaker correlations with RNA-seq data. Thus, while our lower sample size could contribute to lower power in comparison to some studies, we are likely to see stronger correlations with pathology due to our study design. In order to further test the overall consistency of gene expression in these biopsies, we performed differential gene expression and directly compared biopsy tissue with no AD pathology (no β-amyloid or tau, *n* = 32) to biopsy tissue with any AD pathology (either β-amyloid and/or tau, *n* = 74). This analysis only yielded two genes that passed FDR of 0.1 (Supplementary Data 1). Similar analyses based only on β-amyloid (no β-amyloid, *n* = 49 vs. any β-amyloid, *n* = 57) or tau (no tau, *n* = 42 vs. any tau, *n* = 64) yielded 19 and 4 genes passing FDR 0.1 respectively, consistent with the overall low number of significant genes in our correlation analysis (Supplementary Data 1).

**Fig. 2:**
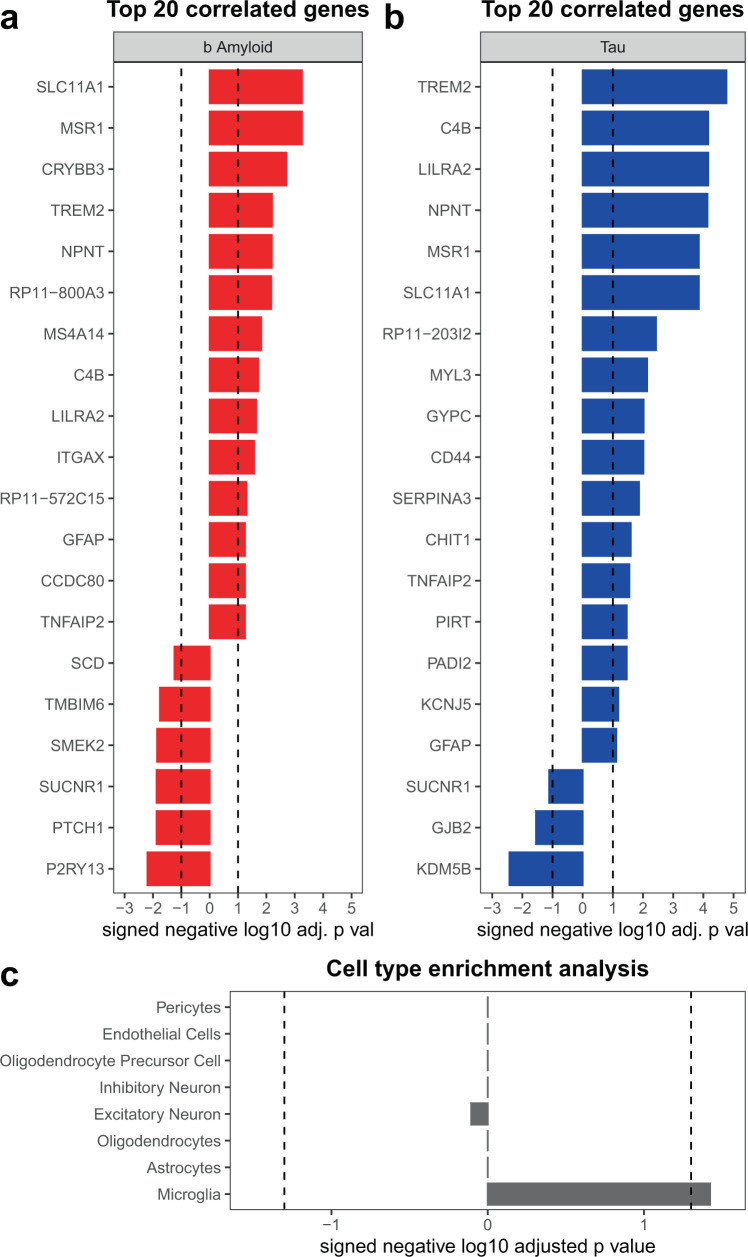
A restricted set of genes correlate with AD pathology in NPH biopsies. Shown are the top 20 genes that correlate with β-amyloid (**a**) and tau (**b**) burden in NPH biopsies (FDR adjusted using the Benjamini–Hochberg procedure across all genes in the transcriptome—dotted line is FDR = 0.1; see Supplementary Data 1 for the full list; all correlations are Spearman’s correlations with two-sided significance). **c** A two-sided Fisher’s exact test confirmed that microglia-specific genes are overrepresented among the genes that individually passed our FDR threshold using human single-nucleus RNA-seq data^21^ (**c** shows Bonferroni adjusted *p* values; dotted line = *p* value of 0.05; See Supplementary Data 1 for numerical values).

The genes that pass FDR threshold in our NPH data are enriched for immune response genes, many of which have been tied to AD. Figure 2 shows the top 20 genes that correlate with β-amyloid plaques and tau burden. A number of immune- or microglia-specific genes were among the top 20 list, including TREM2 and C4B, both of which have been implicated in the immune response in AD^24,25^. These results point to microglia/immune response changes as being important in the very earliest stages of AD pathology and occur before other physiologic changes appear at the bulk RNA-seq level. To confirm this, we determined the level of overlap between our list of genes and lists of genes that characterize specific cell types using human single-nucleus RNA-seq data^26^. A Fisher’s exact test (FET) confirmed that microglia-specific genes are uniquely overrepresented among the genes that individually passed our FDR threshold (Fig. 2c and Supplementary Data 1). It should also be noted that several of the individual genes that reach significance in our analysis are astrocytic. For example, glial fibrillary acidic protein (GFAP) is a reactive astrocyte marker, and CD44, SERPINA3, and C4B have all been associated with disease-associated astrocytes^19,27,28^. While microglia are the predominant cell type represented in our transcriptomic data, an astrocytic response is also clearly present at this early stage of AD pathology.

### AD pathology gene correlations are strongest in patients with subjective cognitive impairment

**Fig. 3:**
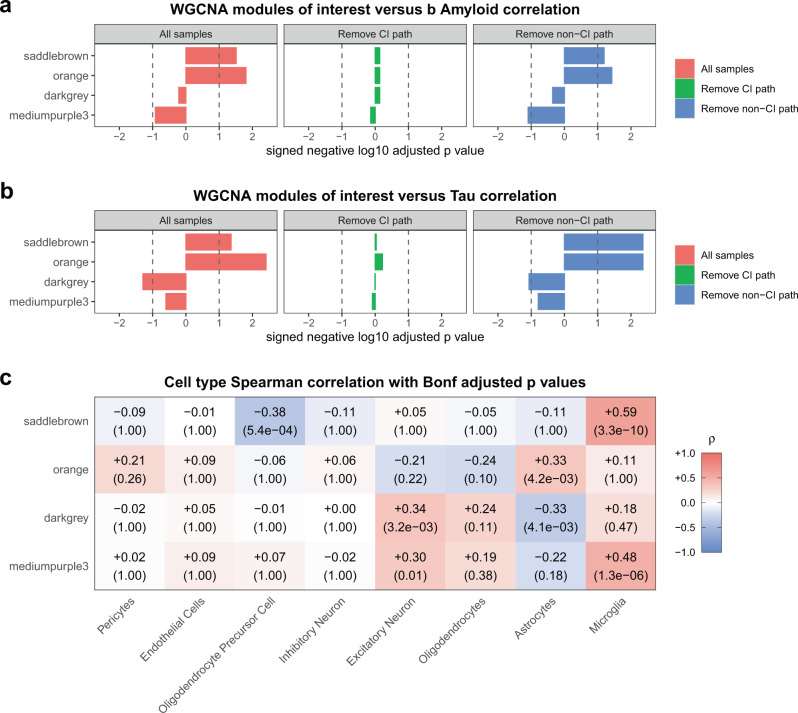
Gene expression modules correlate with AD pathology in the setting of subjective cognitive impairment. **a**, **b** Consistent with the single-gene analysis, WGCNA shows that only three modules (*saddlebrown*, *orange*, and *darkgrey*) correlate significantly with β-amyloid (**a**) and tau (**b**) when all samples (*n* = 106) are considered (red bars on left; see Supplementary Data 2 for the correlations for all WGCNA modules from this analysis and Supplementary Data 3 for the list of genes in key modules in this paper). Subjective cognitive status strongly influences how microglial modules correlate with β-amyloid and tau pathology (green and blue bars). When we removed all samples with AD pathology from our cohort that reported subjective cognitive impairment (leaving samples with AD pathology without subjective cognitive impairment and all non-pathology specimens; the resulting *n* = 66), this abolished the significant correlation of the modules with AD pathology (green bars). In contrast, when we do the converse (i.e., remove all samples with AD pathology from our cohort that do not report subjective cognitive impairment, leaving samples with AD pathology with subjective cognitive impairment and all non-pathology specimens; the resulting *n* = 80), our gene expression modules continue to correlate with AD pathology in the remaining biopsies (blue bars). In fact, one module that was previously not significant becomes significant (*mediumpurple3*). **a**, **b** are FDR adjusted using the Benjamini–Hochberg procedure across all 58 modules in our WGCNA analysis—dotted line in **a**, **b** is FDR = 0.1; all correlations are Spearman’s correlations with two-sided significance. See Supplementary Table 1 for a full breakdown of the cognitive status of all samples in our cohort. **c** The modules in **a**, **b** were correlated with cell-type-specific gene lists (using human single-nucleus RNA-seq data^21^). While *saddlebrown* and *mediumpurple3* are clearly microglial, *darkgrey* and *orange* are more weakly neuronal and astrocytic, respectively, by Spearman’s correlation (each row is separately Bonferroni adjusted, with adjusted two-sided *p* values in parentheses below Spearman’s correlations; see Supplementary Fig. 3 for Fisher’s exact test enrichment of these modules with cell-type-specific gene lists and Supplementary Data 5 for all Spearman’s correlations and enrichment values and *p* values for these modules with different cell types).

Previous work with AD RNA-seq tissue has used gene network analysis to further clarify how groups of genes correlate with various AD traits^1^. When we performed weighted gene co-expression network analysis (WGCNA)^29^ on our NPH data, we identified in total 58 gene co-expression modules, only 3 of which are significantly correlated with either β-amyloid or tau burden (*saddlebrown*, *orange*, and *darkgrey* modules—Fig. 3; see Supplementary Data 2 for full results of WGCNA analysis), consistent with analysis at the single-gene level that a restricted set of genes correlate with AD pathologic traits in this cohort. To further refine our analysis, patient charts were curated for data that would help differentiate the patients by cognitive status. Although rigorous cognitive testing was not consistently carried out, the majority of patients and their families were asked whether they had experienced subjective cognitive impairment during an exam close in time to the biopsy date (see “Methods”). Using this simple metric (yes vs. no), we were able to assign 93 of our sequenced biopsies into these two groups, with 59 replying yes and 34 replying no (the remaining 13 biopsies came from patients where we were unable to locate the answer to this question in the chart; see Supplementary Table 1 for distribution of samples by cognitive status). Patients who reported subjective cognitive impairment had non-significantly higher β-amyloid and tau load than patients who reported no cognitive impairment (*p* value for β-amyloid = 0.21; *p* value for tau = 0.66 by Mann–Whitney *U* test). Interestingly, all of the tau grade 3 biopsies with cognitive information have a history of subjective cognitive complaint. However, these biopsies are so few in number (7) that they do not significantly affect the overall analysis. Interestingly, there is significantly less co-occurrence of β-amyloid pathology and tau pathology in the same biopsy from patients who report no cognitive impairment when compared to patients who do report subjective cognitive impairment (see Supplementary Fig. 2). This suggests that AD pathology may be more widespread in patients who report cognitive impairment (so that we are more likely to see both β-amyloid and tau in a small biopsy), even if the local density of AD pathology in these biopsies is not significantly higher compared to patients who report no cognitive impairment.

Although cognitive status does not predict significantly higher local density of AD pathology in biopsies in our cohort, we did find that the correlations of our modules with AD pathology are being driven by patients who report cognitive impairment. When we removed all samples from individuals with AD pathology who reported cognitive impairment, this abolished the significant correlation of the modules with AD pathology in the remaining biopsies (Fig. 3a, b; see Supplementary Data 2 for full analysis with all WGCNA modules). In contrast, when we do the converse (i.e., remove samples from individuals with AD pathology who did not report cognitive impairment), our gene expression modules continued to correlate with AD pathology in the remaining biopsies. In fact, an additional module reached the significance threshold (*mediumpurple3*; see Supplementary Data 3 for genes in the modules of interest in this manuscript). To further examine the sensitivity of gene correlations with AD pathology to cognitive status, we ran 1000 iterations where we randomly replaced half of the samples with AD pathology in the analysis shown in blue (Fig. 3a, b) with pathology samples in the analysis shown in green (i.e., pathology samples with subjective cognitive impairment are being randomly replaced with pathology samples without documented cognitive impairment). As noted in Supplementary Data 4, this did not statistically change the overall distribution of the burden of pathology in any of the simulations. In contrast, all 4 of our modules fail to pass 0.1 FDR significance in their correlation with β-amyloid and tau for the majority of the simulations. Taken together, these findings indicate that the correlations of these modules with AD pathology are highly sensitive to cognitive status.

**Fig. 4:**
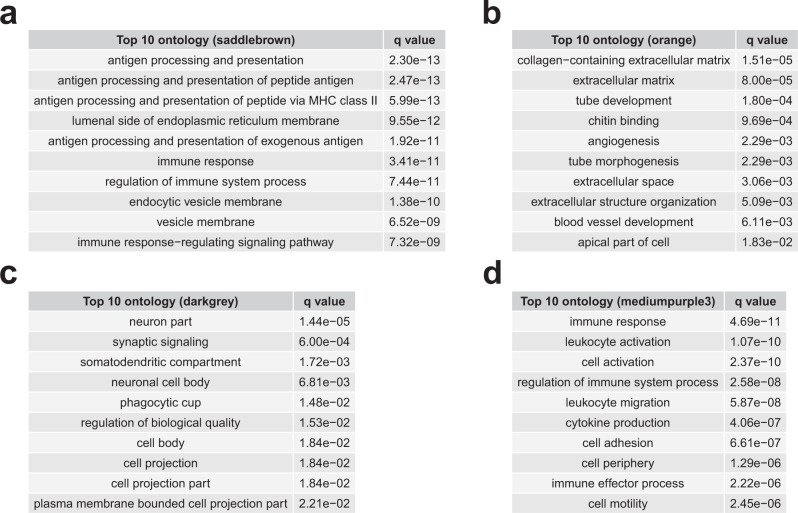
Module ontology analysis suggests cell type specificity. The top 10 ontology groups for *saddlebrown* (**a**), *orange* (**b**), *darkgrey* (**c**), and *mediumpurple3* (**d**) are displayed, along with an FDR-adjusted *q* value that is based on all ontology group one-sided Fisher’s exact test *p* values that were tested (see Supplementary Data 6 for full ontology analysis for these four modules, including all *p* values and FDR-adjusted *q* values).

In an effort to better characterize these modules, we first determined whether these modules correlate with cell-type-specific gene lists. Analysis with gene lists from human single-nucleus RNA-seq data^21^ identified specific cell class assignments for our modules (Fig. 3c), and this is further supported by enrichment analysis (Supplementary Fig. 3 and Supplementary Data 5). These analyses support the view that the *saddlebrown* and *mediumpurple3* modules are predominantly microglial. While the data is somewhat more mixed for *darkgrey* and *orange*, the overall trend is that *darkgrey* is neuronal while *orange* is astrocytic, which is broadly consistent with the positive correlation of the *orange* module and negative correlation of the *darkgrey* module with β-amyloid and tau in the NPH data. Ontology analysis is consistent with these observations (Fig. 4; see Supplementary Data 6 for full ontology analysis results), with *saddlebrown* and *mediumpurple3* characterized by immune response ontology gene sets and *darkgrey* by neuronal gene sets (note that the *orange* module’s cell-type specificity is less clear from ontology analysis).

In our implementation of WGCNA, we allowed for both positive and negative gene correlations within modules (unsigned implementation; see “Methods”). This allowed us to capture more complex changes in physiology within the same module. In addition, using unsigned modules also eliminates the extra analysis step needed to pair signed modules that are anti-correlated with each other. Of the four modules that pass FDR threshold in Fig. 3, *darkgrey* is notable for having the largest fraction of genes that negatively correlate with the PC1 eigengene (37% of *darkgrey* genes correlate negatively with the PC1 eigengene, while the other three modules all have <20% of their genes negatively correlating with PC1; see Supplementary Data 3). For all four of our modules of interest, ontology analysis of positively correlating genes produced similar gene sets as the ontology analysis of the full module (Supplementary Data 6), as well as similar levels of correlations and enrichment with the full module (Supplementary Data 5), consistent with the positively correlating genes dominating the signature of these modules. On the other hand, ontology analysis of negatively correlating genes revealed no significant gene sets for *saddlebrown*, one gene set for *mediumpurple3*, and several gene sets without a clear theme for *orange*. In contrast, ontology analysis for negatively correlating genes in *darkgrey* revealed several significant gene sets related to lipid metabolism (including lipid binding and lipid transporter activity). This suggests that upregulation of lipid metabolism may be an early change that occurs in tandem with early neuronal dysfunction and loss of synaptic/neuronal genes in AD. Lipid metabolism is increasingly recognized as playing an important role in AD pathogenesis^30,31^, and two of the genes from these ontology gene sets (ApoB and PCTP) have recently been implicated in AD through analysis of genome-wide association study data^32,33^. In addition to these changes, we also note several other compensatory genes in *darkgrey* that negatively correlate with the PC1 eigengene, such as HSB1 and neuroglobin, which have both been shown to increase in AD and are thought to be part of the stress response^34–36^. In summary, the *darkgrey* module suggests that we may be capturing early neuronal changes along with compensatory/reactive changes in these biopsies that correlate with increasing pathology most significantly in patients with subjective cognitive impairment. Moreover, this analysis suggests that, while at the single-gene level the changes we are observing are overwhelmingly microglial (Fig. 2), WGCNA is identifying correlating genes from other cell types that may be less significant at the single-gene level.

### NPH modules can be found in other publicly available AD datasets and are enriched for previously identified sets of homeostatic and disease-associated microglial response genes

**Fig. 5:**
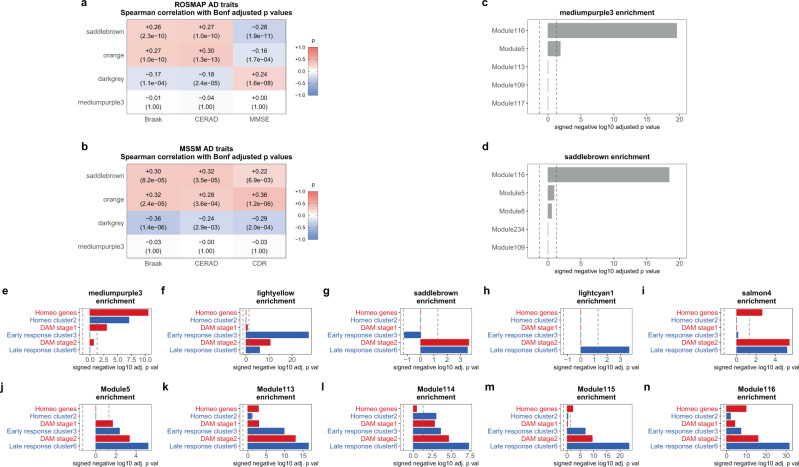
The modules identified in our NPH data also correlate with AD pathology in autopsy cohorts. **a***Saddlebrown*, *orange*, and *darkgrey* correlate with CERAD score, Braak stage, and MMSE score in 596 RNA-seq profiles from the Religious Orders Study and Memory and Aging Project (ROSMAP) Study (representing a range of AD pathologic states)^1^. **b** Similarly, these modules correlate with CERAD, Braak, and CDR score in RNA-seq profiles from frontal cortex in 183 RNA-seq profiles from the MSSM dataset^4^ (all correlations in **a**, **b** are Spearman’s correlations with two-sided significance). **c**, **d** *Saddlebrown* and *mediumpurple3* are both primarily enriched for genes from the same microglial module from Mostafavi et al.^1^, suggesting that microglial genes in our data are correlating differently than in autopsy cohorts (see Supplementary Data 9 for enrichment analysis with two-sided Fisher’s exact test *p* values of *saddlebrown*, *orange*, *darkgrey*, and *mediumpurple3* with all modules from Mostafavi et al.^1^). **e**–**n** The distribution of enrichment for mouse microglial gene lists from Keren-Shaul et al.^37^ (red bars) and Mathys et al.^38^ (blue bars) is more segregated in the top five microglial modules from the NPH data (**e**–**i**) than in the top five microglial modules from Mostafavi et al.^1,39^ (**j**–**n**) (see text for details and discussion and Supplementary Data 8–10 for all Spearman’s correlations and two-sided Fisher’s exact test enrichment *p* values from these panels). For **a**, **b**, each row is separately Bonferroni adjusted, with adjusted *p* values in parentheses below Spearman’s correlations; for two-sided Fisher’s exact test in **c**–**n**, each panel is separately Bonferroni adjusted—dotted line in **c**–**n** is *p* value = 0.05.

We next determined how applicable our findings in NPH tissue are for brains with diagnosed AD. The Religious Orders Study and Memory and Aging Project (ROSMAP) dataset constitutes one of the largest datasets of RNA-seq data from AD autopsy neocortex, and so we sought to examine how well our modules from the NPH tissue correlate with pathologic stigmata of AD in this cohort. RNA-seq and associated metadata for ROSMAP^1^ was downloaded from the AMP-AD Knowledge Portal, and we first regressed out variability in gene expression not correlated with disease-relevant metadata, similar to our processing pipeline for NPH data (see “Methods”). We first sought to determine whether the modules that correlate with β-amyloid and tau in our NPH data correlate with CERAD, Braak, or MMSE score in the ROSMAP data. As shown in Fig. 5a, *saddlebrown* and *orange* both have significant positive correlations with CERAD and Braak stage from ROSMAP, while *darkgrey* negatively correlates with these metadata. In addition, *saddlebrown* and *orange* negatively correlate with MMSE and *darkgrey* positively correlates with MMSE in the ROSMAP data, suggesting that these modules may also be related to cognitive decline (see Supplementary Data 8 for correlations and *p* values). In summary, our glial modules are positively correlating with pathology and cognitive decline while our neuronal module is correlating in the opposing direction, all of which is consistent with known transcriptional changes in the AD RNA-seq literature^1,19,21^. We also examined an additional dataset of frontal cortex RNA-seq data generated at Mount Sinai (MSSM)^4^ and found a similar trend to the ROSMAP data (Fig. 5b).

Strikingly absent from both the ROSMAP and MSSM data is any correlation of *mediumpurple3* with AD pathology or cognition. The *mediumpurple3* is one of the two microglial modules highlighted in Fig. 3, the other being *saddlebrown*. Note that, in our NPH data, the *saddlebrown* module positively correlates with AD pathology, whereas the *mediumpurple3* module negatively correlates with AD pathology. In an effort to characterize these modules further and explain discrepancies with the autopsy-based cohorts, we first examined which of the modules previously identified in the ROSMAP data most overlap with the modules in our NPH data. Interestingly, both *mediumpurple3* and *saddlebrown* overlap the most with the same module from Mostafavi et al.^1^; as seen in Fig. 5c, d, this is module 116 (see Supplementary Data 9 for full analysis of the overlap of our four NPH modules of interest with modules from Mostafavi et al.^1^). Mostafavi et al. identified module 116 as a general microglial module^1^, and the fact that both *saddlebrown* and *mediumpurple3* overlap primarily with 116 suggests that microglial genes are correlating with one another differently in our data than in ROSMAP.

Noting that *saddlebrown* increases with AD pathology and *mediumpurple3* decreases with AD pathology in the NPH data, we next determined whether the *saddlebrown* and *mediumpurple3* modules were associated with known sets of disease-associated microglial genes. Specifically, two recent studies (Keren-Shaul et al.^37^ and Mathys et al.^38^) have identified a transition from homeostasis to a late-stage/disease-associated microglial phenotype in AD mouse models, suggesting that, as AD pathology accumulates, microglia undergo a shift in their transcriptomic profile. As part of this analysis, we generated a list of the top five microglial modules from our WGCNA analysis (based on enrichment with human microglial genes from Mathys et al.^21^) and compared the relative enrichment of these five modules for different disease-stage gene groups from Keren-Shaul et al.^37^ and Mathys et al.^38^ (see Supplementary Data 13 for overlap of these modules with lists from Keren-Shaul et al.^37^ and Mathys et al.^38^). We then compared this analysis with the distribution of different disease-stage gene groups in the top five microglial modules from the ROSMAP analysis^39^. As seen in Fig. 5, microglial modules from our NPH study largely segregate homeostatic and disease-associated gene groups, with no module showing simultaneous enrichment for homeostatic, early-stage, and late-stage genes (and only one module, *salmon4*, showing both homeostatic and late-stage enrichment). Interestingly, the *mediumpurple3* module significantly overlaps with the homeostatic gene lists from these mouse papers. In contrast, the *saddlebrown* module overlaps exclusively with DAM stage 2 and late response genes from these papers. The corresponding negative and positive relationship of these same two modules with AD pathology in the NPH data suggests that these two modules are at least partially tracking an early evolution from homeostatic to late-stage/disease-associated microglia in biopsies with early AD pathology that was first documented in mice.

In contrast, the five microglial modules from Mostafavi et al. are enriched for a broader range of gene lists identified in the mouse literature, and four out of the five are enriched with a list from every category (i.e., homeostasis, early stage, and late stage; Fig. 5j–n; see Supplementary Data 10 for all enrichment values and *p* values). Interestingly, none of the microglial modules from Mostafavi et al. negatively correlate with AD pathology^1^, in contrast to our homeostatic module *mediumpurple3*. We also examined whether the mouse gene groups themselves correlated with AD pathology in NPH, ROSMAP, and MSSM. As seen in Supplementary Fig. 4 and Supplementary Data 11, the homeostatic gene groups defined in mice trend negatively with pathology in NPH data and are either near zero or positively correlate with AD pathology in AD autopsy data. This is again consistent with the finding that homeostatic genes negatively correlate with AD pathology in NPH biopsies and have a more complex relationship with pathology in AD autopsy tissue.

**Fig. 6:**
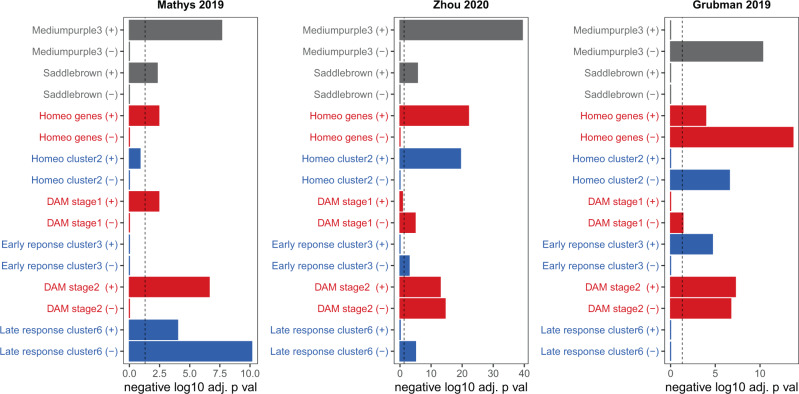
Human single-nucleus microglial RNA-seq data is enriched for our NPH modules and mouse microglial gene lists. For each single-nucleus RNA-seq study, microglial genes that increased (+) or decreased (−) in AD were separately analyzed using two-sided Fisher’s exact test for enrichment for gene sets on the *y*-axis; Supplementary Data 7 has all analyses from this figure. The top two modules in gray (*mediumpurple3* and *saddlebrown*) are NPH modules from this paper; mouse gene lists from Keren-Shaul et al.^37^ (red) and Mathys et al.^38^ (blue) are also shown. Mathys et al.^21^ and Zhou et al.^19^ both show enrichment for lists in the microglial gene set that is increasing in AD, although in both datasets there are some DAM or late-stage gene that are decreasing in AD. Similar to the first two studies, Grubman et al.^22^ also shows a mixed response for DAM genes. Unlike the first two studies, Grubman et al. shows significant overlap of homeostatic gene sets with microglial genes that are decreasing in AD. See main text for discussion. Each study is separately Bonferroni adjusted—dotted line in all panels is *p* value = 0.05.

Although the microglial response in mice differs from humans in many important ways, this data suggests that the transition from homeostasis to a disease-associated phenotype is also occurring to some extent in the NPH data, whereas many of these genes are co-correlating in autopsy data. To further explore this phenomenon, we analyzed single-nucleus RNA-seq data from three different AD tissue studies (Fig. 6 and Supplementary Data 7). Mathys et al.^21^ and Zhou et al.^19^ both analyzed neocortical tissue from AD vs. control. Both studies found that genes that are increasing in AD overlap with both of our NPH microglial modules, as well as several mouse gene lists at a variety of stages (including both homeostatic and DAM2). This is consistent with both homeostatic and late-stage DAM genes increasing in AD autopsy tissue, similarly to the ROSMAP bulk RNA-seq data (Supplementary Fig. 4). Note that Mathys et al. also had a control compared to early AD pathology analysis, but genes changing in this comparison do not overlap significantly with either the *mediumpurple3* module or either of the two homeostatic mouse lists (Supplementary Data 7), suggesting that this particular comparison is still not capturing the early loss of homeostatic genes seen in the NPH biopsies or the mouse literature (see “Discussion”). Grubman et al. performed single-nucleus RNA-seq on entorhinal cortex samples from AD vs. control, and this paper shows that a significant subset of homeostatic genes decline in AD (Fig. 6), which suggests that the autopsy literature is not universally the opposite of the mouse literature with regard to homeostatic genes and AD pathology. It is also interesting to note that late-stage/DAM2 genes are both increasing and decreasing in AD in the single-nucleus RNA-seq literature. Although there are a variety of reasons this may be happening, it has also recently been shown that DAM genes in particular are not well represented in single-nucleus RNA-seq data^40^. Indeed, this could also be a reason why the *saddlebrown* module has weaker associations with the single-nucleus data than the *mediumpurple3* module. We further discuss the Thrupp et al. analysis in the context of the single-nucleus RNA-seq analysis presented here in the “Discussion.”

**Fig. 7:**
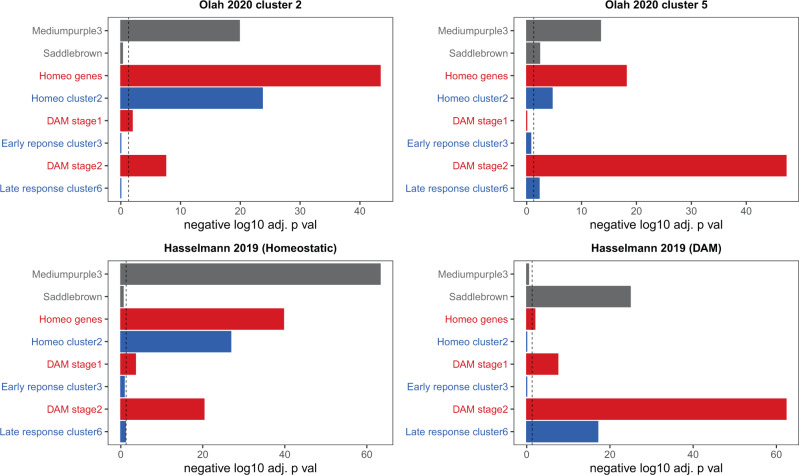
Human single-cell microglial RNA-seq data is enriched for our NPH modules and mouse microglial gene lists. Clusters 2 and 5 are from single-cell sequencing of microglia from autopsy tissue (Olah et al.^20^) and the homeostatic and DAM cluster defining genes from Hasselmann et al.^43^ are from a xenograph mouse model (see text for details). In each panel, the top two modules in gray (*mediumpurple3* and *saddlebrown*) are NPH modules from this paper; mouse gene lists from Keren-Shaul et al.^37^ (red) and Mathys et al.^38^ (blue) are also shown. Cluster 2 from Olah et al. is the cluster from this paper most enriched for homeostatic genes from the mouse literature, and cluster 5 is most enriched for DAM stage 2 genes. Nevertheless, both clusters are also enriched for genes from opposing gene lists (i.e., cluster 2 is enriched for DAM stage 2 genes and cluster 5 is enriched for homeostatic genes), and both are enriched for *mediumpurple3*. The homeostatic and DAM xenograph clusters are enriched exclusively for *mediumpurple3* and *saddlebrown*, although even here there is some crossover enrichment with mouse lists (i.e., DAM stage 2 gene enrichment with the homeostatic cluster). See main text for discussion and Supplementary Data 7 for all analyses with these datasets, including the analysis displayed in this figure. Each analysis in this figure uses a two-sided Fisher’s exact test and is separately Bonferroni adjusted—dotted line in all panels is *p* value = 0.05.

Single whole-cell sequencing of microglia presumably circumvents the problems identified by Thrupp et al., and we compared our modules to two recent single-cell studies to further examine the applicability of our findings to the AD autopsy literature. Olah et al. recently identified 9 distinct microglial clusters from a set of AD and non-AD tissue^20^, and the two most relevant clusters for our findings are shown in Fig. 7 (see Supplementary Data 7 for analysis with all clusters from this paper)^20^. Cluster 2 is the cluster most enriched for genes from homeostatic groups, which Olah et al. also defined as a homeostatic group using a Naive Bayes classifier. Interestingly, Olah et al. found that cluster 2 is the cluster most enriched for genes that positively correlate with β-amyloid and tau and is also the cluster with the second strongest enrichment for genes that positively associate with clinical AD^20^. This is consistent with the finding repeated several times in this manuscript that homeostatic genes positively correlate with AD traits in autopsy datasets. The cluster most enriched for DAM stage 2 genes is cluster 5, which they identified as the cluster with the strongest positive association with genes that correlate with clinical AD and the second strongest positive association with genes that correlate with β-amyloid (second to cluster 2). Note that, although clusters 2 and 5 are the clusters most enriched for homeostatic and DAM stage 2 genes, respectively, both clusters are also enriched for some mouse gene lists from other categories (Fig. 7). In general, the clusters from Olah et al. are enriched for a broad range of microglial gene lists from the mouse literature (Supplementary Data 7). This suggests that multiple clusters of microglia contribute to the homeostatic or DAM signal in this tissue, and may partially explain why these gene lists co-correlate in late-stage neocortical AD autopsy tissue (Fig. 5). Alsema et al. also recently profiled single-cell microglia^41^ and found no relevant differences in microglial composition or gene expression between AD and controls. While this could be for a variety of reasons (as discussed in their paper), we note here that the microglial clusters identified in their manuscript show less overlap with our NPH modules as well as all six mouse microglial gene lists in comparison to the microglial clusters from Olah et al. (see Supplementary Data 7). Thus, the Alsema et al. analysis is less consistent with both our data and the mouse data in comparison to the Olah et al. analysis.

The transcriptomic response of xenograph human microglia in AD mouse models has recently been studied, and we examined how our modules overlap with and possibly help inform this data. Mancuso et al. recently studied the human microglial response to β-amyloid injection in an in vivo rodent central nervous system (CNS) environment^42^. This acute treatment led to a microglia population shift away from a homeostatic cluster and toward a cytokine response cluster, and genes that define these clusters overlap significantly with the homeostatic and DAM mouse lists, respectively, as well as with the corresponding modules from this paper (i.e., the homeostatic module overlaps with *mediumpurple3* and the cytokine response cluster overlaps with *saddlebrown*; Supplementary Data 7). Hasselmann et al. recently investigated how human microglia respond in an AD transgenic mouse model^43^. The authors identified a homeostatic cluster (which did not significantly change in their AD model), as well as a cluster that was enriched for murine DAM genes (which increased in population in AD transgenic mice). The Hasselmann homeostatic and DAM clusters are strongly enriched for *mediumpurple3* and *saddlebrown*, respectively, but have some cross-overlap with opposing lists from the mouse literature (Fig. 7). If one assumes that human brain tissue is the actual ground truth, then this suggests that our NPH data may help further clarify which genes identified in common between mouse studies and human xenograph studies are important in the response to early-stage AD pathology.

### NPH modules correlate with microglial histologic features

**Fig. 8:**
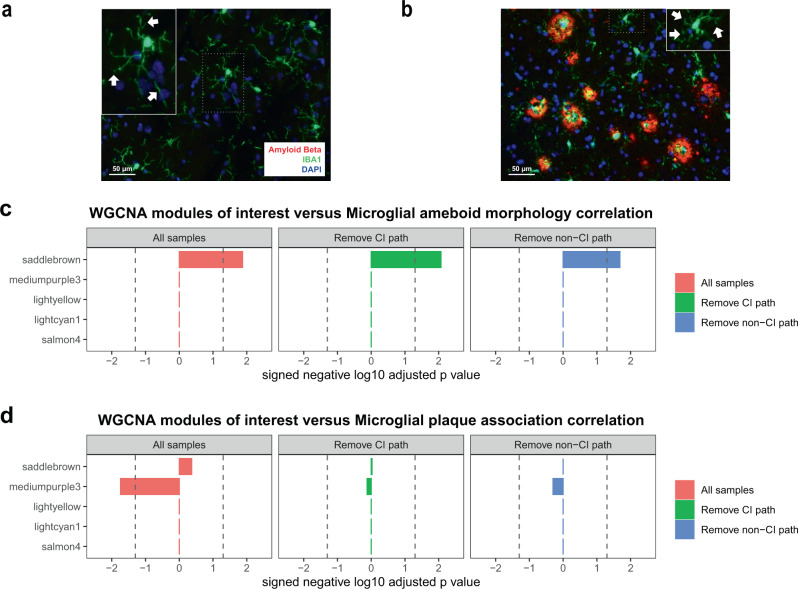
Our microglial modules correlate with microglial morphology and location. We performed β-amyloid/IBA-1 dual staining on selected cases from our cohort. **a** Cases without β-amyloid showed microglia with longer processes (white arrow; microglial cell in inset is blow-up from area demarcated with dotted line), while **b** cases with β-amyloid pathology tended to show more ameboid, activated microglia. We stained 59 biopsies for β-amyloid/IBA-1 and this data is used to calculate correlations in **c**, **d**. As noted in the “Results” section, microglial ameboid morphology moderately correlated with plaque area in the same biopsy (*r* = 0.3; two-sided *p* value = 0.01; ameboid morphology was measured using the compactness metric in Cellprofiler—see “Methods” for details). **c**, **d** We assessed the Spearman’s correlation with two-sided significance of the top five microglial modules shown in Fig. 5e–i for microglial ameboid morphology (measured using the compactness metric in Cellprofiler—see “Methods” for details) (**c**) and microglial β-amyloid plaque association (**d**). Only *saddlebrown* significantly correlates with microglial ameboid morphology, and this is seen whether or not samples with AD pathology come from patients who report cognitive symptoms (the green and blue bars represent groups formed using the same methodology as in Fig. 3a, b). In addition, only the *mediumpurple3* module significantly (and negatively) correlates with microglial plaque association, suggesting that plaque association correlates with loss of homeostatic genes. When we eliminate samples with AD pathology based on cognitive status, we lose significance for *mediumpurple3*, which suggests that this correlation is somewhat weak in this dataset, and also that both sample groups are likely contributing to the overall significance of this finding in the full dataset. In **c**, *n* = 59 for all samples (red bars), *n* = 35 in the Remove CI path group (green bars), and *n* = 42 in the Remove non-CI path group (blue bars); in **d**, *n* = 29 for all samples, 11 in the Remove CI path group (green bars), and *n* = 18 in the Remove non-CI path group (blue bars). The three panels in **c** and the three panels in **d** are each separately Bonferroni adjusted—dotted line in all panels is *p* value = 0.05; see Supplementary Data 12 for all Spearman’s correlations, *p* values, and adjusted *p* values from this figure.

Finally, in an effort to elucidate the relationship between microglia, β-amyloid pathology, and our modules, we performed IBA-1/β-amyloid dual staining in NPH biopsies that we sequenced and correlated our microglial gene expression modules with microglial morphology in the same biopsies. Microglia in biopsies with β-amyloid plaques tended to be more compact, with an activated, ameboid-like morphology (Figs. 8a and 6b), and microglial ameboid morphology moderately correlated with plaque area in the same biopsy (*r* = 0.3; *p* value = 0.01; *n* = 59; ameboid morphology was measured using the compactness metric in Cellprofiler—see “Methods” for details). Ameboid morphology also correlated exclusively with the *saddlebrown* module after Bonferroni adjustment (Fig. 8c; see Supplementary Data 12 for all correlations and *p* values). In addition to *mediumpurple3*, we also assessed the correlation of the other three microglial modules from our NPH data shown in Fig. 5. Interestingly, even though several other modules are enriched for disease-associated genes identified in the mouse AD literature, only *saddlebrown* significantly correlates with microglial ameboid morphology. The correlation of *saddlebrown* with microglial morphology is also seen whether or not samples with AD pathology come from patients who report cognitive symptoms. This suggests that microglial activation and upregulation of genes in the *saddlebrown* module is an initial response that does not immediately relate to cognitive impairment. Also notable is that *mediumpurple3* is not significant in any comparison, suggesting that loss of homeostatic genes is not related to microglial ameboid morphology in these biopsies.

We also examined the relationship between microglia and plaques in our biopsies, and the relationship with module behavior. Specifically, we looked at the degree of microglial plaque association and normalized by the total plaque area in each analyzed image (Fig. 8d, see “Methods” for details). Interestingly, the *mediumpurple3* module is the only significant module and negatively correlates with microglial plaque association, suggesting that plaque association correlates with loss of homeostatic genes. In contrast, the other microglial modules (including *saddlebrown*) show no significant correlation with plaque association. When we eliminate samples with AD pathology from patients either with or without subjective cognitive impairment, we lose significance for *mediumpurple3*, which suggests that this correlation is weak in this dataset and also that both sample groups are likely contributing to the overall significance of this finding in the full dataset. Noting that *saddlebrown* correlates with morphology but not with plaque association, we further examined microglial morphology both near and further away from plaques and noted that, in slides with β-amyloid, both sets of microglia have similar ameboid morphology (Supplementary Fig. 5). Thus, it appears from our data that morphologic changes in microglia occur in the local vicinity of plaques, regardless of whether or not the microglia are physically overlapping the plaque. Although this does not clarify the exact mechanism for how our modules relate to these histologic features, this dissociation between morphology and plaque association is consistent with different modules correlating with these two phenomena. Finally, microglial numbers did not significantly correlate with any of the five microglial modules nor do they correlate with amyloid plaques in our biopsies (*r* = −0.12; *p* value = 0.35; *n* = 59), consistent with previous observations that amyloid plaque deposition happens earlier than increases in microglial number^44^, as well as side-by-side comparisons of single-nucleus RNA-seq data showing that the expansion in microglial numbers seen in AD mouse models is not as prominent in human AD tissue^19^. One caveat to these observations is that the number of a motile cell type such as microglia counted in a single section of tissue may only loosely correlate with the average number of microglia in the surrounding tissue. Thus, we may not be sufficiently powered to pick up correlations between modules and overall microglial number using this imperfect measure. In summary, the associations of *saddlebrown* and *mediumpurple3* with microglial morphology and plaque association do not seem to be sensitive to patient cognitive status, in contrast to the association of these modules with accumulating AD pathology. This is consistent with the view that an initial microglial response to AD pathology is eventually associated with accumulating pathology, non-microglial cell responses, and cognitive dysfunction (see “Discussion”).

## Discussion

In this study, we have used cortical biopsies from hydrocephalus patients to examine changes in gene expression that accompany early-stage AD pathology. Our rationale for examining gene expression and AD pathology in these biopsies is: (1) This cohort represents a large sample of relatively young patients, many of whom have early-stage AD pathology^8,12^. (2) The tissue in this cohort is also free of gene expression changes that accompany end-of-life hypoxia/apoptosis state, as well as any changes in RNA caused by post-mortem degradation. (3) All cognitive data curated from the patients’ charts represent the cognitive state relatively close to the time of tissue acquisition (see “Methods”). The primary findings from this effort are: (1) A limited set of microglial and non-microglial modules correlate primarily with AD pathology in patients with subjective cognitive impairment. (2) The microglial modules identified in this analysis correlate in a coherent way with homeostatic and disease-associated gene expression lists from the mouse model literature and with microglial subtypes from the human single-cell literature. (3) In contrast to the existing AD autopsy literature, these microglial modules replicate both the decrease in homeostatic genes in parallel with an increase in late-stage disease-associated genes seen in the mouse AD literature. (4) When analyzed in comparison to IBA1-β-amyloid dual-stained sections, our microglial modules correlate with plaque association and microglial morphology, and this change is not sensitive to cognitive status. Taken together, these data suggest that an initial microglial response is associated with accumulating pathology, non-microglial cell responses, and patient-reported cognitive status.

As noted in the “Results” section, we have identified a gene expression module enriched for murine disease-associated microglial genes that positively correlates with AD pathology (*saddlebrown*) and a module enriched for murine homeostatic microglial genes that negatively correlates with AD pathology (*mediumpurple3*), and in aggregate, this is more consistent with the existing mouse literature than publicly available AD autopsy datasets^1–4,19,21,22^. Although it is not entirely clear why our data more closely aligns with the mouse AD literature than existing autopsy datasets, there are several important differences between our data and human AD autopsy data that may influence the differences we are seeing. One obvious difference is that our tissue was obtained in a different way from AD autopsy tissue. Specifically, our tissue was removed from living cortex while the patient is under anesthesia, as opposed to being acquired after patient death. In this regard, the lack of any post-mortem artifact in our data makes it more similar to tissue obtained from laboratory mice, and one could speculate that post-mortem factors may partially obscure the AD-associated microglial response. Although this presents an intriguing possibility for how our data may be more similar to the mouse literature, an alternative (and not mutually exclusive) explanation is that hypoxia and/or sepsis at the end of life could be affecting the CNS inflammatory response in a way that obscures or further obscures the AD-associated microglial transcriptomic response. With regard to the downregulation of homeostatic genes, we did identify one autopsy dataset (in entorhinal cortex) that shows a subset of homeostatic genes that decreases in AD, which somewhat mitigates a purely artifactual explanation for why the majority of the autopsy datasets show no change or an increase in homeostatic genes in AD. Note, however, that the entorhinal cortex study (Grubman et al.^22^), similarly to the neocortical studies of Zhou et al.^19^ and Mathys et al.^21^, also shows a mix of increasing and decreasing DAM genes in AD, with an almost even split for the DAM stage 2 genes from Keren-Shaul et al.^37^. As noted earlier, one possible explanation for this comes from a recent study by Thrupp et al.^40^ which suggests that DAM genes in particular may be missing from single-nucleus RNA-seq, which only captures nuclear RNA. In fact, this study directly compared the genes depleted in single-nucleus RNA-seq vs. single-cell RNA-seq to the data from Mathys et al. and showed that most of the DAM genes highlighted in the Mathys et al. paper were the subset of DAM genes that had relatively higher expression in the nucleus. Note that Thrupp et al. did not include disease tissue in their analysis, and it is not clear whether DAM genes are more or less expressed in the nucleus in AD in comparison to their data. How much the Thrupp et al. finding may affect the analysis of nuclear RNA-seq studies presented here is unclear, but this could be an important factor explaining discrepancies between the single-nucleus RNA-seq data and the bulk RNA-seq data presented in this manuscript.

Of course, we should also note that our biopsies represent an earlier stage of pathology than most autopsy cohorts. Indeed, one major caveat to this study is that these patients do not have AD per se, but rather AD pathology. As mentioned in the “Introduction” section, NPH patients with AD pathology are less responsive to shunting^8,13^ and are at an increased risk of developing AD^6^, which supports studying this cohort as a group that may represent a pre-AD population. In addition, AD is clinically defined as AD pathology in the setting of cognitive impairment on a neuropsychology exam^45^, and our changes in gene expression are most robust in patients who report subjective cognitive impairment, which suggests that what we are seeing in these biopsies is indeed relevant for the earliest stages of AD. However, our metric for measuring cognitive decline is based on subjective reporting by the patient during a contemporaneous clinical exam. We used this metric because standardized neuropsychology testing was not carried out on all patients, while documentation of the patient-reported subjective cognitive impairment was found in a large majority (93) of the patient files for the biopsies sequenced in this study. Although subjective cognitive decline correlates with a patient’s psychometric assessment^46^, it is not as precise as formal neuropsychology testing. Another major caveat is that AD pathology is common in elderly subjects without cognitive impairment, many of whom die before ever developing AD dementia^16–18^, and the biological meaning of AD pathology in subjects without cognitive impairment is still an area of investigation^47–49^. This is particularly relevant because the metric for measuring cognition in this study is not precise, so we cannot definitively say which patients in our study are pre-AD. One possible interpretation of our data is that AD pathology is associated with a more robust biological effect in patients with subjective cognitive decline because these patients are moving into the earliest stage of AD, and the relative lack of significant genes in our analysis overall is a reflection of this early point in the disease trajectory. However, a more careful study of this patient population with formal neuropsychology testing and longitudinal follow-up would be necessary to definitively say which patients will progress to clinical AD.

Note that other studies have included early AD groups, although these groups often have more pathology on average than our cohort. For example, Mathys et al.^21^ has an early AD cohort in their study. However, 8 of the 15 subjects in this group are Braak stage 5, which would indicate significant cortical tau pathology in half of their early AD subjects (although this is less pathology than in their late-stage subjects). As noted in Fig. 5, microglial gene groups from the mouse literature co-correlate in the ROSMAP modules. This mixing of microglial responses in the ROSMAP microglial modules may partially explain the relative lack of correlation of these modules with AD pathology in Mostafavi et al.^1^. However, as noted in their paper, their module 116 did correlate with AD pathology when analyzed in the data of Zhang et al^3^. Although untangling the reasons for all of these discrepancies is outside the scope of this study, we note here that the general phenomenon of co-correlation raises the possibility that a general microgliosis and increased microglia infiltration late in AD may cause the apparent reversal of the decline in homeostatic genes. However, note that an upregulation of homeostatic genes in human AD autopsy tissue is seen in several single-nucleus and single-cell RNA-seq studies analyzed in this paper. This eliminates the possibility that increased homeostatic gene expression in later-stage AD is entirely due to changes in cellular composition and points to an actual change in microglial gene transcription. Confounding all of these analyses is the additional issue that all of these autopsy cohorts are also older than ours. For example, the mean age of the ROSMAP dataset is 88.7 years^1^, the mean age of the MSSM dataset is 84.7 years^4^, and the Mathys et al. cohort has an average age of 86.7 years for the AD group and 87.1 years for the non-pathology group^21^.

Nevertheless, our data suggest that the downregulation of homeostatic genes in conjunction with an increase in late-stage/disease-associated genes is occurring at the earliest stages of AD pathology in neocortex. The implication of this work is that the AD mouse literature may be modeling the earliest stages of the microglial response to AD pathology in a more faithful way than previously recognized. The Grubman et al. study is the most similar to our cortical NPH biopsies. Although this is only one study, one could speculate that entorhinal cortex and neocortex may have an initial common microglial response to AD pathology that diverges in neocortex as pathology accumulates. Future work will be needed to better determine the similarities and differences between the microglial response in entorhinal cortex and neocortex.

It should be noted that three of the top five microglial modules in our WGCNA analysis by enrichment (*lightyellow*, *lightcyan1*, and *salmon4*) do not correlate with any of the pathologic metrics in this paper, despite being significantly enriched for several groups of disease-associated genes from the mouse literature (Fig. 5). This highlights an obvious point that the microglial response in mice is not exactly the same as in humans. However, it also suggests that the data in this paper can serve as a conceptual bridge between some of the early responses seen in the mouse literature and the human AD literature. While there will always be interspecies differences in these comparisons, the closer similarity of our data to the mouse literature suggests that our data may help clarify which aspects of mouse biology may be accurately modeling the early microglial response in humans.

It should also be noted that while the Keren-Shaul et al. study used an APP-based model (5×FAD)^37,50^, Mathys et al. used the CK-p25 model, which shows elevated β-amyloid as well as neurofibrillary tangles^38,51,52^. Similarities and differences in the immune response to different neurodegenerative disease pathologies is an area of active research, and future work will be needed to determine which findings from this paper are specific for different AD pathologies or generalizable to other neurodegenerative diseases. Indeed, although we have highlighted the microglial response in this paper, there is no a priori reason to assume that the microglial response in our data is pathogenic or even specific for AD. This highlights a related point that our work also identifies non-microglial genes as being important in the earliest stages of AD pathology. Although significant genes at the individual level are mostly microglial (Fig. 2), it should be noted that several of the individual genes that reach significance in our analysis are astrocytic. For example, GFAP is a reactive astrocyte marker, and CD44, SERPINA3, and C4B have all been associated with disease-associated astrocytes^19,27,28^. While microglia are the predominant cell type represented in our transcriptomic data, an astrocytic response is also clearly present at this early stage of AD pathology even at the single-gene level. This observation is further supported by our WGCNA analysis, which identifies an astrocytic (*orange*) and neuronal (*darkgrey*) module in addition to our two microglial modules. One might therefore look at the *orange* and *darkgrey* modules for astrocytic and neuronal genes that are involved in early cognitive decline in the setting of a prolonged microglial response.

Human tissue samples are often acquired for clinical and circumstantial reasons beyond the control of the researcher, and the tissue used in this study also has important limitations for similar reasons. Most obviously, all of the patients in this study have the co-morbitidy of hydrocephalus. Hydrocephalus biopsies are collectable from alive individuals and therefore do not present some of the limitations of analyzing post-mortem brain tissue. However, the presence of hydrocephalus can also be a confounding factor and it is not easy to disentangle what effect this might have on gene correlations with AD pathology. Never the less, there are several reasons to hypothesize that the effect of hydrocephalus may be minimal when comparing the data in this study to other gene expression studies on human AD autopsy brain tissue. First, AD is rarely pure in a pathologic sense. In recent years, there has been a growing appreciation that dementia in the elderly is often due to several co-morbid conditions in any individual, as vascular disease, Lewy body dementia, and TDP-43 pathology are frequently seen in AD autopsy cohorts^53–55^. Thus, pure AD is actually less common among patients with dementia than mixed pathology. Previously reported gene correlations with AD pathology were presumably found in spite of these common confounders, and there is no a priori reason to expect hydrocephalus to uniquely affect these correlations more than other common confounders. In addition, it should again be noted that, although we have found far fewer changes in gene expression in comparison to previous studies of AD brain tissue, we have also found other changes (i.e., a loss of homeostatic genes) that are not found in AD autopsy cohorts but are found to some extent in AD animal models. Although one could theorize that findings in this study are discordant with the AD autopsy literature due to hydrocephalus, the alignment of these findings with the AD animal literature strongly suggests that we are observing the same phenotypic change that has been documented multiple times in AD animal models.

In conclusion, this study identifies a restricted set of genes that correlate with early AD cortical pathology and subjective cognitive impairment and points to future directions for research into how microglia may mediate early cognitive decline. In addition, this work identifies NPH patients with AD pathology as a possible pre-AD population that may benefit from early intervention in AD clinical trials. Finally, this study suggests that this patient population may be particularly interesting to study prospectively, and future studies will seek to link these gene expression modules with subsequent cognitive decline or cognitive resilience.

## Methods

### NPH biopsy sample collection and histopathology studies

This study was reviewed and approved by the Columbia University Review Board (study plan IRB-AAAT7985), and all relevant ethical regulations have been followed. This study is a retrospective study that uses residual tissue samples not required for clinical diagnosis and associated clinical and demographic data. At the time of surgery, a portion of tissue was frozen and deposited in the Bartoli Brain Tumour Bank at Columbia University. Patients undergoing surgery signed an umbrella consent form, providing consent to use the remaining biopsies and body fluids for research. A portion of tissue was submitted for clinical analysis. After completion of the clinical tests, any residual tissue can be released by the tissue bank for research purposes. Tissue and clinical data used in this study were provided de-identified by the tissue bank.

NPH was originally defined by ventricular dilation with normal CSF pressure, with a classical clinical triad of imbalance/ataxia; cognitive impairment, particularly short-term memory decline; and urinary incontinence^56,57^. The usefulness of the concept of NPH has been more recently challenged^58,59^ due to the fact that the symptoms in this clinical triad are common in the elderly population at large^59^; that (conversely) all three symptoms may not actually occur in all patients diagnosed with NPH^59,60^; and that the CSF pressure in NPH patients can actually be variably elevated^10,61^, suggesting a chronic deficit in CSF resorption. Not surprisingly, NPH may sometimes arise secondarily to known medical conditions that affect CSF resorption, leading to the concept of secondary NPH, to be distinguished from iNPH^9,10,58,59^. Patients suspected of having NPH can be clinically stratified into probable NPH vs. possible NPH, depending on how well the patient’s symptoms match the appropriate clinical picture and whether any co-morbidities may also be accounting for the patient’s clinical and imaging findings^60^. We performed RNA-seq on 106 biopsies from NPH patients, 90 of which fell within the definition of probable or possible iNPH, according to the criteria of Relkin et al.^60^ (16 biopsies came from patients with a known benign lesion near the cerebral aqueduct that may have contributed to the patient’s chronic hydrocephalus, which would qualify as secondary NPH). Our cohort of 106 samples has an average age of 74.9 years (standard deviation 8), with 42 females and 64 males. In all cases, patients were shunted for chronic hydrocephalus by the same surgical team, and biopsies were taken from either frontal cortex (middle frontal gyrus at coronal suture) or parietal cortex (~4 cm off midline in parietal lobe, just above parieto-occipital junction). The decision to shunt/biopsy in frontal or parietal cortex was made by the surgeon based on cosmetic considerations; in total, approximately 2/3 of our cohort have frontal biopsies and 1/3 have parietal biopsies. Cortical biopsies were divided in the operating room immediately after removal, and half of each biopsy was frozen in liquid nitrogen while the other half was formalin fixed and paraffin embedded for subsequent pathology diagnosis (Fig. 1).

In addition to a hematoxylin and eosin stain, immunohistochemistry was performed with antibodies against tau (AT8 at 1:200 dilution; Thermo Fisher; Catalog # MN1020), β-amyloid (6E10 at 1:200 dilution; BioLegend; Catalog # 803003), α-synuclein (KM51 at 1:40 dilution; Leica; Catalog # NCL-L-ASYN), and TDP-43 (C-terminal rabbit polyclonal at 1:500 dilution; Proteintech; Catalog # 12892-1-AP). All slides were counterstained with hematoxylin. Immunostaining was performed in the Ventana automated slide stainer without manual antigen retrieval and was detected using the Ventana ultraView Universal DAB Detection Kit (Tucson, AZ) as recommended by the manufacturer. Patients in this study had variable amounts of β-amyloid and tau pathology and no α-synuclein or TDP-43 pathology or any other visible diagnostic abnormality on hematoxylin and eosin staining. β-Amyloid plaques were counted per square mm; in slides with enough tissue, three fields were averaged together, whereas in slides with less tissue, the largest number of possible fields were counted. For tau quantification, we devised a rating scale to grade the minimal degree of tau pathology seen in NPH biopsies (see Supplementary Fig. 1). Grade 0 was given to biopsies with no tau pathology. Grade 1 was given to biopsies that have any tau pathology at all, usually one or more dystrophic neurites, but do not make criteria for Grade 2. Grade 2 was given to biopsies that have at least one tau-positive neuron or neuritic plaque, but do not make criteria for Grade 3. Grade 3 was reserved for biopsies with tau pathology evenly distributed throughout the biopsy.

Note that a Braak stage cannot be assigned to these biopsies, as Braak staging is a global assessment of tau pathology that takes into account the presence and density of tau in multiple regions^62^. Although tau begins to spread broadly into neocortex at Braak stage 4, AD tau pathology shows natural variability from case to case, and occasional focal staining of higher Braak stage areas can be observed at earlier Braak stages^62^. Thus, in the absence of additional anatomical data Braak staging is not possible. Moreover, it should be emphasized that the primary purpose of the tau grading system devised in this manuscript is to measure the local density of pathology and compare it to local changes in gene expression. All of these considerations apply to Thal staging of β-amyloid as well (i.e., Thal stage requires a global assessment of β-amyloid burden)^63^. In this case, note that β-amyloid appears initially in neocortex (Thal stage 1), so all of our biopsies with β-amyloid would qualify as Thal stage 1, and we cannot stage higher using only neocortical tissue.

Additional patient data (sex, age, NPH diagnosis, and subjective cognitive status on intake exam) were gathered from the medical record. Cognitive exam data were gathered from patient medical exams as close to the time of biopsy as possible. If possible, an exam note was located where the patient was asked whether they had experienced subjective cognitive impairment. Using this simple metric (yes vs. no), we were able to assign 93 of our sequenced biopsies into yes or no, with 59 replying yes and 34 replying no (the remaining 13 biopsies came from patients with no clear answer from the medical record). The average time between exam and biopsy among all 93 samples was 120 days. Patients who reported subjective cognitive impairment had non-significantly higher β-amyloid and tau load than patients who reported no cognitive impairment (*p* value for β-amyloid = 0.21; *p* value for tau = 0.66 by Mann–Whitney *U* test). Interestingly, all of the tau grade 3 biopsies with cognitive information have a history of subjective cognitive complaint. However, these biopsies are so few in number (7) that they do not significantly affect the overall analysis (see Supplementary Table 1 for distribution of samples by cognitive status).

### NPH sample RNA sequencing and data preprocessing

RNA was extracted from biopsy samples using the miRNeasy Mini Kit (QIAGEN; Cat No./ID: 217004), which purifies total RNA including miRNA (note that our library prep protocol selects poly(A) coding mRNA). RNA integrity was measured on an Agilent Bioanalyzer, and samples with RNA integrity number (RIN) values ≥6 were selected for sequencing. RNAs were prepared for sequencing using the Illumina TruSeq mRNA Library Prep Kit, and samples were sequenced with Illumina HiSeq 2000, 2500, and 4000 (potential batch effect attributable to different sequencers was regressed out along with other confounding variables using surrogate variable analysis; see below), and all samples underwent single-end sequencing to 30 M read depth. The quality of all fastq files was confirmed with FastQC v 0.11.8^64^. Fastq files that passed quality check were further mapped to Genome Reference Consortium Human Build 37 (GRCh37) reference genome with STAR^65^. Output BAM files from STAR were further processed with featureCounts^66^ to obtain raw counts for each gene of all the sequenced samples.

Genes with <5 counts in at least 90% of all samples were first filtered out from the raw count matrix, followed by variance stabilizing transformation (VST) on filtered counts utilizing the *varianceStabilizingTransformation* function from DESeq2 R package^67^. VST is a widely applied strategy that transforms data from a distribution of fluctuating variance into a new distribution with nearly constant variance in order to facilitate downstream analysis^68^. After VST, surrogate variable analysis (SVA)^23^ was used to identify variation in gene expression not attributable to β-amyloid load or tau load. Specifically, a full model (with primary variables to be kept) and a null model (with intercept only) were built using the model.matrix() function, and then sva() function from sva r package was applied to determine surrogate variables (SVs) from the VST-processed gene expression matrix, in which sva function parameter dat was assigned with the gene expression matrix, mod as the full model, mod0 as the null model and method = irw. The identified SVs, which represent known and unknown confounding variables in our dataset, were later regressed out with *removeBatchEffect* function from limma R package^69^. The filtered, VST-processed, and surrogate variable regressed count matrix was used for all downstream analyses in this manuscript. As an additional exercise, we also attempted to regress out confounding variables individually. To do this, the NPH expression matrix was first quantile-normalized, followed by log2 transformation, and then the batch effect was removed through the ComBat R function from the sva package, and finally age, gender, and RIN were regressed out using the removeBatchEffect R function from the limma package. This method did not yield any significant genes at an FDR threshold of 0.1, confirming the relatively minimal transcriptomic changes in this tissue cohort. This also highlights the need to regress out both known and hidden confounders in our dataset in order to detect the relatively minimal changes in gene expression that we do find. Finally, we also performed differential gene expression analysis on the NPH count data as an additional exercise and used DESeq2^67^ to do the following comparisons: (1) No AD pathology (no β-amyloid or tau, *n* = 32) vs. any AD pathology (either β-amyloid and/or tau, *n* = 74), (2) No β-amyloid pathology (*n* = 49) vs. any β-amyloid pathology (*n* = 57), and (3) No tau (*n* = 42) vs. any tau (*n* = 64). These analysis yielded 2, 19, and 4 genes passing FDR 0.1, respectively (Supplementary Data 1). These results further confirm the overall consistency of gene expression signatures in these biopsies and supports our view that this tissue is from patients with the earliest stages of AD pathology, before more extensive pathophysiologic changes have occurred.

For our analysis below, we combine RNA-seq data from all biopsies to achieve higher statistical power; this analysis is effectively investigating changes in gene expression shared by two areas of neocortex related to early AD pathology. This power is necessary to detect the relatively subtle changes in gene expression accompanying these early changes in AD pathology (see below). Analyzing frontal and parietal areas separately showed that both analyses trended in a way similar to the combined dataset (see Supplementary Data 1).

### RNA-seq data analysis and module characterization

For single-gene analysis, we calculated the Spearman’s correlation between β-amyloid and tau burden and individual genes using the *cor.test* function in R. The *p* values were further Benjamini–Hochberg (BH) adjusted across all the genes in the dataset using the *p.adjust* function in R. To generate gene expression modules, we utilized WGCNA to identify gene co-expression modules^29^, on the default (unsigned) setting, with softPower = 7 and minModuleSize = 20. The eigengene for each WGCNA module was correlated with β-amyloid and tau burden and eight cell-type-specific signatures from the human single-nucleus RNA-seq literature^21^ (Inhibitory neurons, Excitatory neurons, Oligodendrocyte precursor cells, Oligodendrocytes, Astrocytes, Microglia, Endothelial cells, and Pericytes). Note that for all correlations of WGCNA eigengenes with other gene lists (including the cell-type-specific gene lists above and mouse and human microglial subtypes below), the WGCNA eigengene is correlated with the mean gene expression vector of these various lists. While the eigengene is the preferred way of capturing the variance of highly correlated WGCNA gene modules^29^, there is no a priori reason to assume that the eigengene will capture the majority of the variation in gene lists that are not formed though measuring intercorrelations and thus may not be as highly intercorrelated as WGCNA modules. Thus, for cell-type and microglial gene lists we use the mean gene expression vector as a more holistic measure of variation of these gene lists.

To identify cell class-specific genes from single-nucleus RNA-seq data from the Mathys et al. study^21^, we first used edgeR to calculate differential expression among all pairs of broad cell classes annotated in the study; each individual nucleus was treated as a sample in edgeR, and un-normalized count values were used. For each of these pairs, we then selected cell class-specific genes as those having at least 1.5 positive fold change (FC) between the class of interest and the mean transcripts per million (TPM) value of all other classes in a pairwise fashion, with the added condition that a given candidate gene mean TPM value should be larger or at least equal to 20. The selected genes were further ranked in descending order based on FCs, from which the mean expression of the top 500 genes were used for correlation analysis.

In addition, FET was performed to check the enrichment of cell type for the group of individual genes that passed FDR threshold or specific modules of interest. For enrichment analysis, all cell-type-specific genes that passed the filtering criteria for FPKM (fragments per kilobase per million) and FC were selected for the test. The FET *p* values were Bonferroni corrected across all cell types for each comparison. The ontology analysis was performed with all the genes in a given module using over-representation analysis under the gene set analysis tab from ConsensusPathDB web tool^70^. All the level 2 and 3 ontology groups, including all three categories (molecular function (m), biological process (b), and cellular component (c)), with at least two shared genes with the test module were selected and enrichment *p* values were calculated through FET. The *q*-values were FDR adjusted across all the selected ontology terms within each category (m, b, or c). All selected ontology groups were further ranked in ascending order based on *q*-values, and the top 10 most significantly enriched pathways are listed in Fig. 4.

Note that, for this study, we have employed an FDR threshold of 0.1 when screening large numbers of hypotheses to identify genes or modules of interest (i.e., genes or WGCNA modules that correlate with AD pathology or ontology groups that are enriched for module genes). Throughout the rest of the manuscript, tests of hypotheses about these genes/modules have employed a Bonferroni correction with a *p* value threshold of 0.05.

### Comparison of NPH modules with AD autopsy datasets

We compared NPH datasets with two publicly available AD autopsy datasets:

1. The ROSMAP Study (Synapse ID: syn3219045)
  RNA-seq raw counts in total 596 human subjects were obtained from paired-end fastq files using STAR + featureCounts pipeline for pair-ended reads. The raw counts matrix was filtered and VST transformed as described above. Finally, variation in gene expression not attributable to Braak stage, CERAD score, and MMSE was regressed out using SVA, similar to our processing pipeline for NPH data.
2. The Mount Sinai Brain Bank Study (Synapse ID: syn3159438)
  We downloaded 183 bulk RNA-seq raw count tables from human brain samples from Brodmann area 10. Samples were preprocessed the same way as our NPH samples and the ROSMAP data, and SVA was used to regress out variability not attributable to Braak stage, CERAD score, and CDR score.

### Effects of cognitive status on NPH AD traits

In Supplementary Fig. 2, patients who report subjective cognitive impairment are compared to patients who report no cognitive impairment. The cognitive information of each subject was obtained as described above. Mann–Whitney *U* test was first performed to examine the difference in average β-amyloid and tau load between subjects who report cognitive impairment and subjects who report no cognitive impairment. To further explore the distribution of these variables, we first performed FET on whether tau and β-amyloid significantly co-occur in biopsies from patients with reported cognitive impairment vs. biopsies from patients who report no cognitive impairment. We next performed a Mantel–Haenszel test of whether the odds ratios between the two groups were different (Supplementary Fig. 2). In Fig. 3a, b, we asked whether samples with AD pathology from either group were significantly driving the correlation between the modules and AD pathology. To do this, we first removed all samples with AD pathology from our cohort where patients reported cognitive impairment (our Remove CI path group, *n* = 66). Next, we removed all samples with AD pathology where patients who reported no cognitive impairment (our Remove non-CI path group, *n* = 80). Note that, in both groups, all biopsies without AD pathology were included. Finally, we ran 1000 iterations where half of the samples with AD pathology from the Remove non-CI path group (20 out of 40) were randomly selected to be replaced by another randomly selected 20 samples with pathology from the Remove CI path group to form an artificial Remove non-CI path group (i.e., pathology samples with subjective cognitive impairment are being randomly replaced with pathology samples without documented cognitive impairment). Mann–Whitney test of β-amyloid or tau between the real and artificial groups and correlations of module eigengenes with β-amyloid or tau in both groups were performed for each iteration and *p* values from these tests were recorded. The correlation *p* values were further BH adjusted across all WGCNA modules. At the end of all iterations, the number of times out of 1000 iterations (1) Mann–Whitney test *p* value was <0.05 for β-amyloid or tau and (2) the BH adjusted correlation *p* values that passed significance threshold (0.1) for any of the four modules in Fig. 3 was reported. As noted in Supplementary Data 4, this did not statistically change the overall burden of pathology in any of the simulations. In contrast, all four modules of interest fail to pass 0.1 FDR significance in their correlation with β-amyloid and tau for the majority of the simulations.

### Microglia activation stage- and subtype-specific gene identification

Microglia activation stage-specific genes from mouse studies were obtained from two separate datasets. We used three sets of genes reported in Keren-Shaul et al.^37^ (Homeostatic genes, DAM stage 1, and DAM stage 2) and three sets of genes reported in Mathys et al.^38^ (Homeostatic cluster 2, Early response cluster 3 (the primary early response cluster), and Late response cluster 6). We defined cell-type-specific genes using comparisons done in these manuscripts. Specifically, homeostatic genes from Keren-Shaul et al. were defined as differentially expressed (DE) genes with a minimum twofold increase in expression compared to Tg 5×FAD samples and FDR <0.1. The DAM stage 1 and DAM stage 2 genes were filtered through similar criteria except that the DE genes were calculated based on the comparison between these two DAM stages. For gene lists from Mathys et al. (i.e., clusters 2, 3, and 6), cluster-specific genes were defined as genes upregulated in a given cluster in comparison to either of the other two clusters and not downregulated in either of these two comparisons. The significance of these comparisons was defined as absolute value of the corrected *z* score >1.25, which is equivalent to an FDR corrected *p* value >0.1. Mouse gene symbols were converted to human gene symbols with R biomaRt package for comparison with the data in this paper.

### Immunofluorescence staining and imaging

Immunofluorescence was performed on cortical biopsies for β-amyloid and IBA-1 to visualize microglial morphology and microglial plaque association. Fixed, paraffin-embedded tissue was sectioned at 7 µm, and slides were submerged in two washes of Histo-clear II (National Diagnostics HS-202) for 10 min each, before being washed in the following series for 1 min each: 100% ethanol, 100% ethanol, 70% ethanol, and MQ water. They were then washed 3 times in Tris-buffered saline (TBS) for 5 min each. Antigen retrieval was performed by incubating the slides in citrate buffer for 25 min in a microwave set to 400 Watts. The slides in citrate buffer were rested at room temperature for 30 min before a 5-min wash with MQ water, two 5-min permeabilization washes in TBS with 0.25% Triton X-100 (ACROS Organics), and a 5-min wash in TBS. The sections were blocked with 10% donkey serum (Abcam ab7475) and 1% bovine serum albumin (BSA; Sigma-Aldrich A7284) in TBS for 1 h at room temperature. A primary antibody solution was created in 1% BSA in TBS, with the addition of mouse polyclonal anti-β-amyloid (1:200; Cell Signaling Catalog # 15126, lot 1) and rabbit polyclonal anti-IBA1 (1:500; Wako Catalog # 019-19741, lot ptr2404). The sections were stained overnight in the primary antibody solution at 4 °C, after which they underwent three 5-min washes in TBS. A secondary antibody solution was created in 1% BSA in TBS, with the addition of Alexa Fluor 555-conjugated Donkey Anti-Mouse (1:1000; Invitrogen A-31570, lot 1850121) and Alexa Fluor 488-conjugated Donkey Anti-Rabbit (1:1000; Invitrogen A-21206, lot 1981155). The sections were incubated for 1 h at room temperature in the secondary antibody solution, after which they underwent three 5-min washes in TBS. To block autofluorescence, Sudan Black B (EMS 21610) solution at 0.1% m/v in 30% MQ water and 70% ethanol was placed on the sections for 20 min, after which they underwent three 5-min washes in TBS. Nuclei were labeled with 4,6-diamidino-2-phenylindole (1:4000 from stock; Invitrogen D1306) in TBS for 5 min, before three 1-min washes in TBS. Slides were mounted with Vectashield (Vector H-1000).

Cellprofiler was used to perform image analysis on the immunohistochemistry-stained slides^39,71^. IBA1 staining area to determine microglia density was identified with thresholding based on the background variance. Individual Iba1-stained microglial cells were identified with thresholding after successive edge-preserving smoothing to remove stray processes, and the microglia cell shape was traced by enhancing microglia process shapes and thresholding the resulting image. Plaques were identified with thresholding based on the background variance. Microglial infiltration of plaques was determined from the total Iba1 staining area overlapping plaques for a sample, normalized by total plaque area. Microglial compactness was calculated on individual Iba1-stained cells as a measurement output defined by Cellprofiler as the mean squared distance of the object’s pixels from the centroid divided by the area. Calculated this way, this metric is lower for more compact cells; in Fig. 8, we invert this value so that our modules positively correlate with compactness.

### Reporting summary

Further information on research design is available in the Nature Research Reporting Summary linked to this article.

## Supplementary information

Supplementary Information

Description of Additional Supplementary Files

Supplementary Data 1

Supplementary Data 2

Supplementary Data 3

Supplementary Data 4

Supplementary Data 5

Supplementary Data 6

Supplementary Data 7

Supplementary Data 8

Supplementary Data 9

Supplementary Data 10

Supplementary Data 11

Supplementary Data 12

Supplementary Data 13

Reporting Summary

## Data Availability

The RNA-seq data generated in this study have been deposited in the synapse.org (Sage Bionetworks) database under 10.7303/syn21898410 [https://www.synapse.org/#!Synapse:syn21898410/wiki/603968]. The RNA-seq data and associated metadata are available under restricted access due to the fact that this data is derived from human tissue samples, and access can be obtained by making a formal request through the synapse.org database. Data from all figures is available in Supplementary Data and Source data files. ROSMAP data^1^ and MSSM data^4^ were both downloaded from synapse.org (Sage Bionetworks) database. ROSMAP data (Synapse ID: syn21589959) was downloaded from https://www.synapse.org/#!Synapse:syn21589959 and MSSM data (Synapse ID: syn7416949) was downloaded from https://www.synapse.org/#!Synapse:syn7416949. Source data are provided with this paper.

## Source data

Source Data
