# Supplementary material for: An immune response characterizes early Alzheimer’s disease pathology and subjective cognitive impairment in hydrocephalus biopsies
Source: Nat Commun. 2021 Sep 27;12:5659. doi: 10.1038/s41467-021-25902-y (PMC8476497; doi:10.1038/s41467-021-25902-y)
Supplement: Supplementary file 1 — Supplementary Information [file 41467_2021_25902_MOESM1_ESM.pdf]

Supplementary Information

The biopsies in our cohort have minimal tau pathology on immunohistochemistry

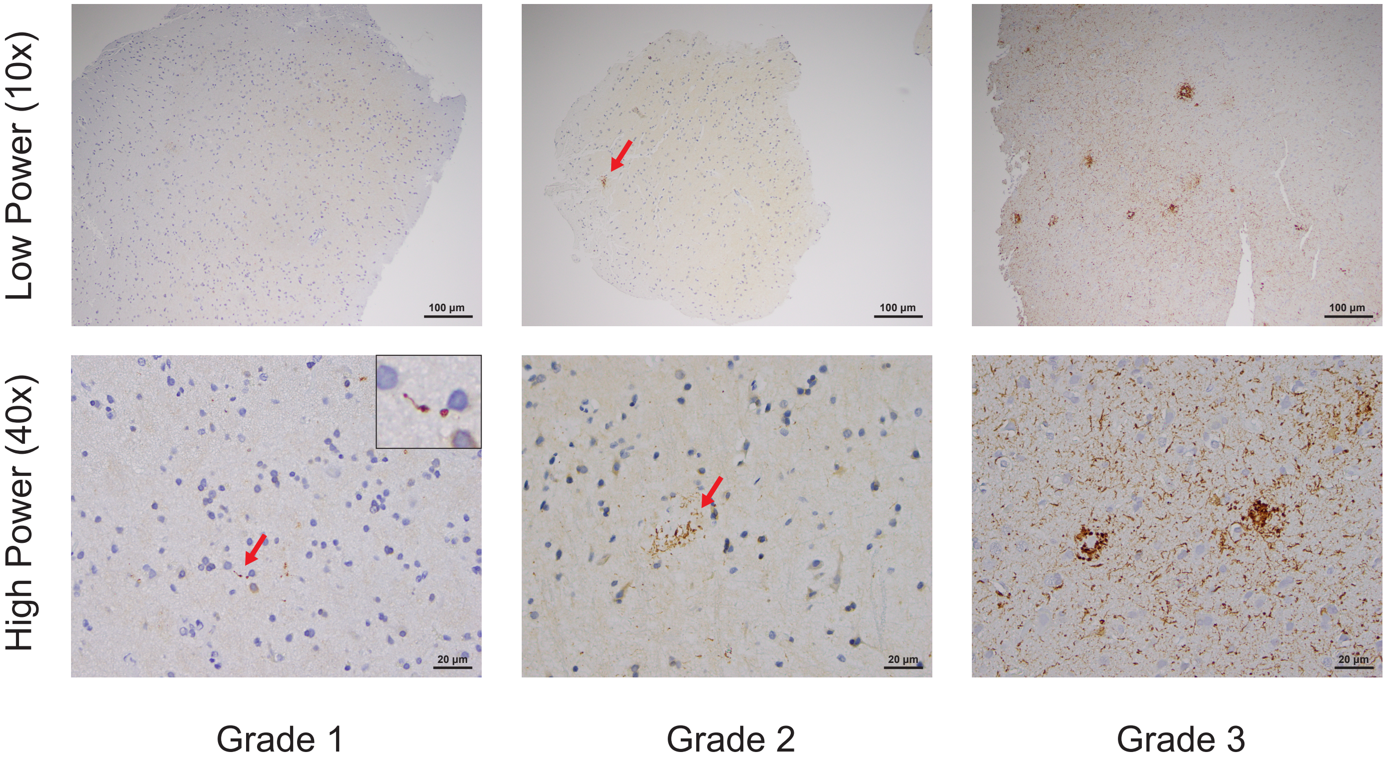

Supplementary Figure 1 - We devised a rating scale to grade the minimal degree of tau pathology seen in NPH biopsies. All panels display tau immunohistochemistry of NPH biopsies at low-power (10x magnification, upper row) or high-power (40x magnification, lower row). Grade 0 was given to biopsies with no tau pathology. Grade 1 (left panels) was given to biopsies that have any tau pathology at all, usually one or more dystrophic neurites, but do not make criteria for Grade 2 (middle panels). Grade 2 was given to biopsies that have at least one tau-positive neuron or neuritic plaque, but do not make criteria for Grade 3 (right panels). Grade 3 was reserved for biopsies with tau pathology evenly distributed throughout the biopsy. It is difficult to appreciate tau pathology at low-power in grade 1 slides (upper left panel), and high-power is required to see single dystrophic neurites (lower left panel - red arrow, see blow-up insert). In grade 2 slides, one can usually see neuritic plaques at low-power (middle upper panel, red arrow), as well as at high-power (middle lower panel, red arrow). Grade 3 slides have obvious tau pathology at all magnifications. Note that it is not possible to assign a Braak stage to these cases based on a single biopsy (see Methods for full discussion of this issue). Note that tau staining was done on all biopsies in our study (n = 106). Supplementary Figure 2 shows the distribution of tau scores in our cohort.

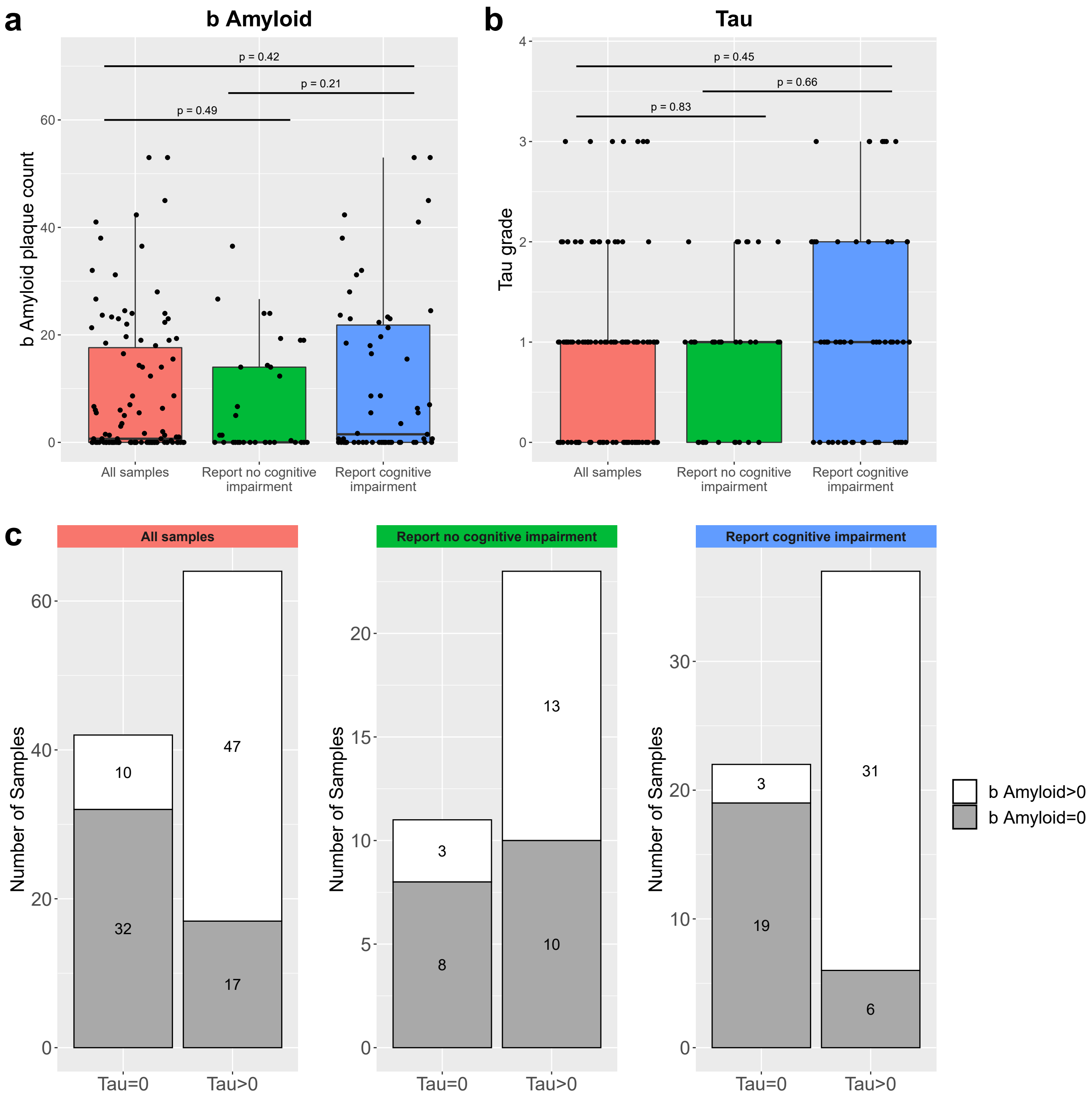

Supplementary Figure 2 - Cognitive status minimally influences AD pathologic burden in this cohort. Panels A and B: Patients who report cognitive impairment have a non-significant trend for higher levels of β-amyloid and tau on biopsy when compared to patients who report no cognitive impairment (box plots bounded by first and third quartile; thick black bars show median and whiskers show 1.5 inner quartile distance above third quartile; p-value for β-amyloid = 0.21; p-value for tau = 0.66 by two-sided Mann Whitney U test). Panel C: Despite a non-significant difference in overall AD pathology, patients who report cognitive impairment are more likely to have β-amyloid and tau in the same biopsy (Fisher exact test for presence of both β-amyloid and tau in biopsies from patients that report cognitive impairment two-sided p-value = 1.827633e-07, for patients that report no cognitive impairment two-sided p-value = 0.152; Mantel Haenszel test for whether odds ratios between the two groups are different: two-sided p-value = 8.881858e-07). Note that the presence of 9 samples with no cognitive information and zero tau score in the full cohort make the overall average tau value in panel B (all samples) lower than the average value between patients that report cognitive impairment and

patients that report no cognitive impairment. Individual data points for this figure are available in the Source Data File.

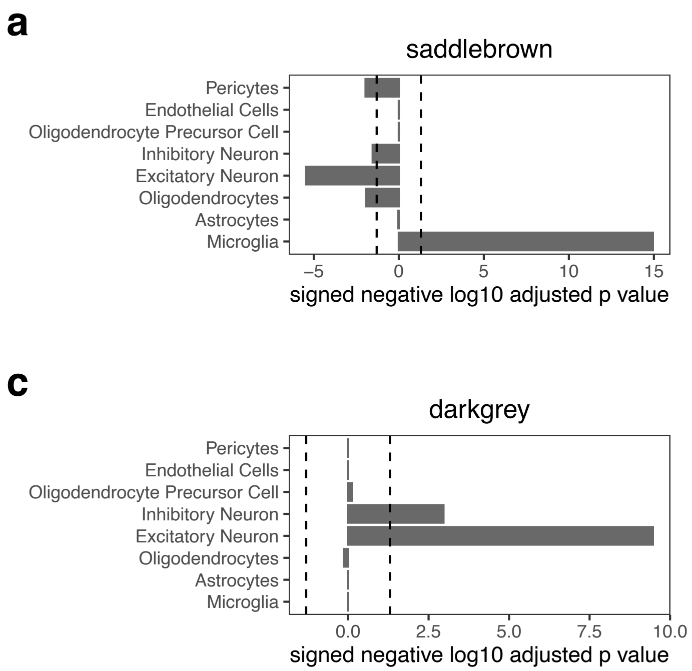

Supplementary Figure 3 – Shown is the enrichment for each of the four modules in Figure 3 with eight cell-type specific signatures from the human literature [1] using the two-sided Fisher's exact test; see Supplementary Data 5 for full Spearman's correlation and enrichment analysis of these modules with these cell types. Each panel is separately Bonferroni adjusted; dotted line in all panels is p-value = 0.05.

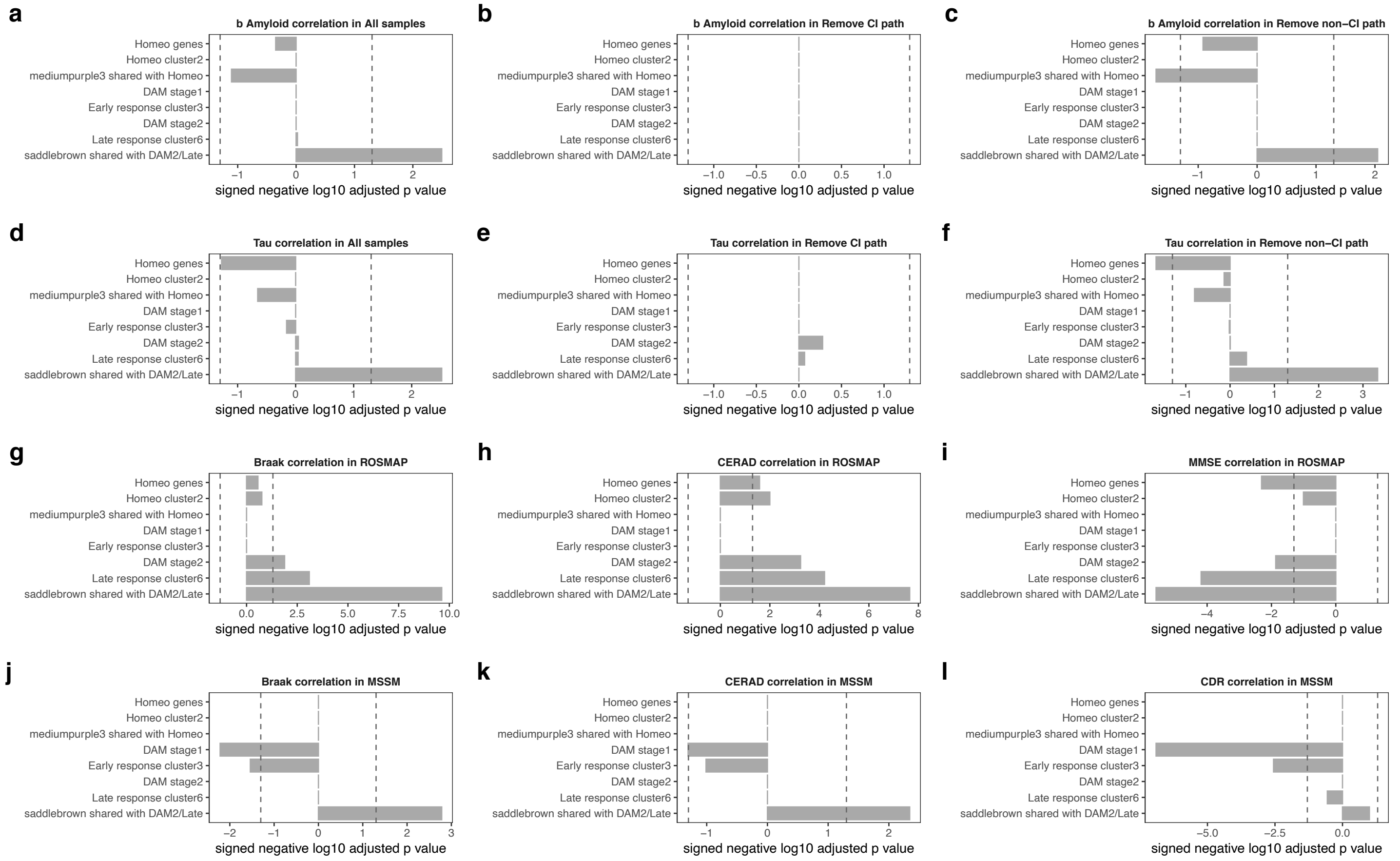

Supplementary Figure 4 – Mouse microglial genes variably correlate with AD pathology in NPH and autopsy tissue. Shown are Spearman's correlations with two-sided significance of three sets of genes reported in Keren-Shaul, et al. [2] (Homeostatic genes, DAM stage1 and DAM stage2), and three sets of genes reported in Mathys, et al. (2017) [3] (Homeostatic cluster 2, Early response cluster 3 (the primary early response cluster), and Late response cluster 6) with β-amyloid and tau in NPH samples (panels A and D) and our remove CI path (panels B and E) and remove non-CI path (panels C and F) groups from Figure 3. Spearman's correlation of these mouse modules with AD traits in ROSMAP (panels G, H, and I) and MSSM (panels J, K, and L) are also shown. In each panel is also shown the correlation of the subset of genes in *mediumpurple3* that overlap with the homeostatic lists from Keren-Shaul, et al. [2] or Mathys, et al. (2017) [3], and the correlation of the subset of genes in *saddlebrown* that overlap with DAM stage 2 or late response cluster 6 from Keren-Shaul, et al. [2] and Mathys, et al. (2017) [3] (see Supplementary Data 13 for overlap of these modules with lists from Keren-Shaul, et al. [2] and Mathys, et al. (2017) [3]). Note that in NPH data, homeostatic genes negatively correlate with β-amyloid and tau, while late stage/disease associated gene lists from mice positively correlate (although not significantly) with β-amyloid and tau. Not surprisingly, homeostatic and late stage/DAM stage 2 genes that overlap with *mediumpurple3* and *saddlebrown* respectively correlate stronger with AD pathology in the NPH biopsies. Autopsy datasets offer a more complex figure, with ROSMAP data showing positive correlations of both homeostatic and late stage/disease associated genes with AD traits, similarly to what is reported in Zhou et al. [4]. The MSSM dataset offers a somewhat different picture, with early response and DAM stage 1 genes negatively correlating with AD pathology, while homeostatic and late stage/DAM stage 2 genes show little correlation. Note, however, that although the late stage/DAM stage 2 lists do not correlate with pathology as a whole in the MSSM dataset, the subset of these genes that overlap with *saddlebrown* do strongly correlate with AD traits in the MSSM dataset, consistent with an ongoing microglial response that may not completely correspond to the mouse literature. Each panel is separately Bonferroni adjusted p-values are shown; dotted line in all panels is p-value = 0.05. See Supplementary Data 11 for all of the Spearman's correlation values, p-values, and adjusted p-values in this figure.

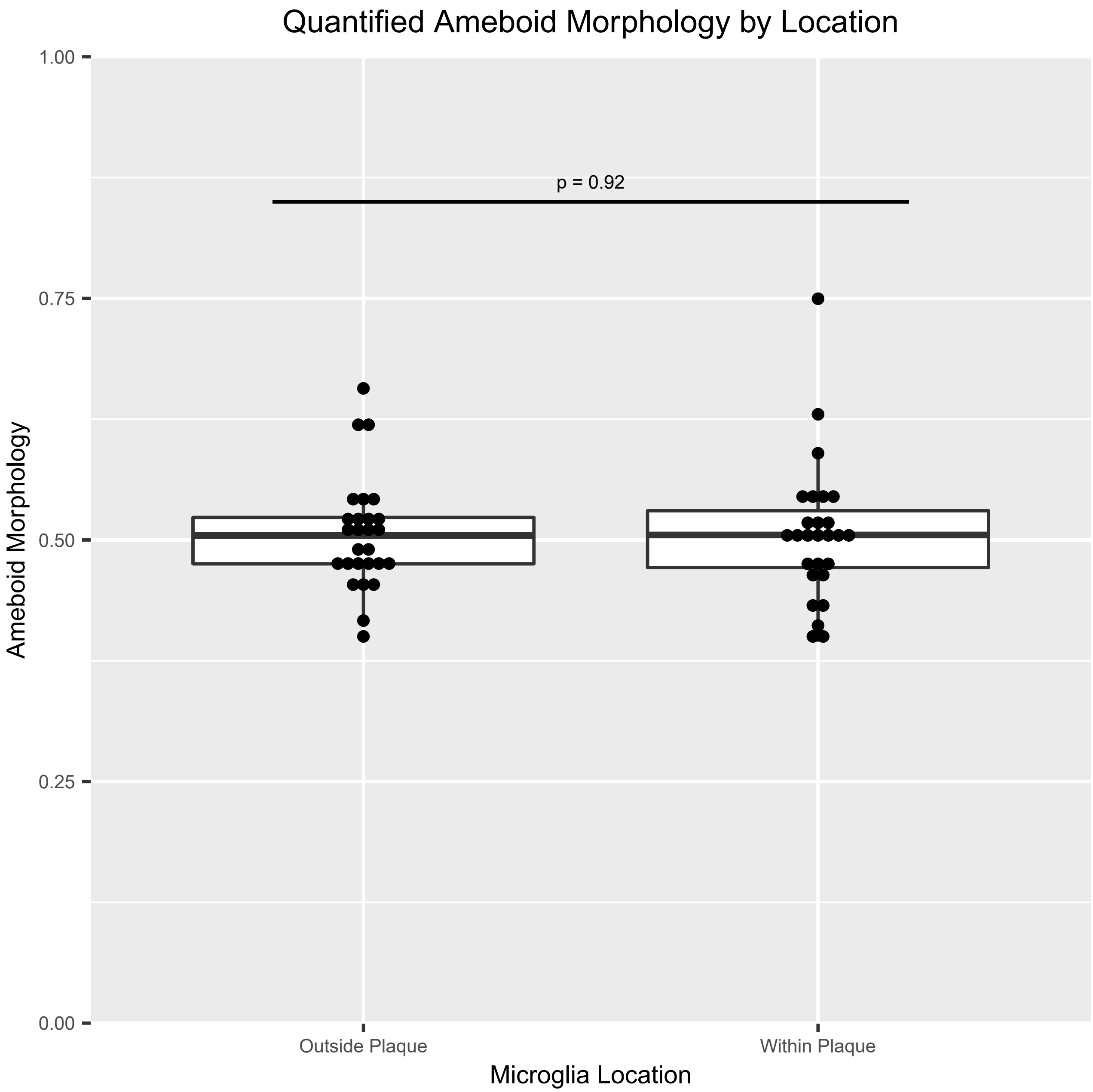

Supplementary Figure 5 – Ameboid morphology of microglia is similar both inside and outside of β-amyloid plaques. We analyzed ameboid morphology (the compactness metric with Cellprofiler – see Methods) for microglia that were overlapping with plaques and compared to microglia in the same slide that did not overlap plaques. Although compactness correlates with β-amyloid plaque count overall (see Results section), within slides with β-amyloid plaques there is no difference in this metric based on microglia location. n = 27 slides analyzed, p-value = 0.92 using two-sided paired Wilcoxon signed rank test; box plots bounded by first and third quartile; thick black bars show median and whiskers show 1.5 inner quartile distance below and above the first and third quartiles. Individual data points for this figure are available in the Source Data File.

b Amyloid plaque count	CI path	Non-CI path	N/A	All
none	25	18	6	49
< 10	13	5	6	24
≥ 10	21	11	1	33
**Total**	59	34	13	106

Tau grade	CI path	Non-CI path	N/A	All
0	22	11	9	42
1	19	16	4	39
2	11	7	0	18
3	7	0	0	7
**Total**	59	34	13	106

Supplementary Table 1: Number of patients with each grade of b Amyloid and Tau pathology among cohorts with cognitive impairment (CI path) and without cognitive impairment (Non-CI path). N/A corresponds to patients for whom an exam note indicating cognitive impairment status could not be located.

Supplementary Data Legends:

Supplementary Data 1 - list of all genes that correlate with β-amyloid and tau in the full dataset, also correlations with frontal samples only and parietal samples only, along with p-values and adjusted p-values using the Benjamini-Hochberg procedure. For the top 100 genes that correlate with β-amyloid in the full dataset, the Spearman's correlation coefficient of these genes with β-amyloid in frontal and parietal samples are themselves highly correlated, with a Spearman's correlation of r=0.8691309, and two-sided p-value<2.2E-16 across all 100 genes. Both frontal and parietal samples are equally similar to the full dataset as well, with a Spearman's correlation of r = 0.9893544, and two-sided p-value<2.2E-16 when the correlation of these 100 genes with β-amyloid are compared between the full dataset vs. frontal samples, and r=0.9297355, and two-sided p-value<2.2E-16 when the comparison is between the full dataset and parietal samples. A similar relationship holds with tau; for the top 100 genes that correlate with tau in the full dataset, the correlation coefficient of these genes with tau in frontal and parietal samples have a Spearman's correlation of r=0.7948915, and two-sided p-value <2.2E-16. When the correlation of these 100 genes with tau are compared between the full dataset vs. frontal samples, r = 0.9769622, and two-sided p-value<2.2E-16, and when the correlation of these 100 genes with tau are compared between the full dataset vs. parietal samples, r=0.9037152, and two-sided p-value<2.2E-16. Also included in this table file are the p-values and adjusted p-values that correspond to a two-sided Fisher's exact test enrichment for cell-type specific genes among the genes that individually past FDR 0.1 threshold in the full dataset, using human single-nucleus RNA-seq data [1]. Finally, in order to further test the overall consistency of gene expression in these biopsies, we performed differential gene expression, and directly compared biopsy tissue with no AD pathology (no β-amyloid or tau, n = 32) to biopsy tissue with any AD pathology (either β-amyloid and/or tau, n = 74). This analysis only yielded two genes that passed FDR of 0.1. Similar analyses based only on β-amyloid (no β-amyloid, n = 49 vs. any β-amyloid, n = 57) or tau (no tau, n = 42 vs. any tau, n = 64) yielded 19 and 4 genes passing FDR 0.1 respectively. See main text for discussion and analysis.

Supplementary Data 2 – Spearman's correlation of all WGCNA modules with β-amyloid and tau pathology in all samples, in the remove CI-path group, and in the remove non-CI path group from Figure 3, along with two-

sided p-values and adjusted p-values using the Benjamini-Hochberg procedure.

Supplementary Data 3 – shown are all genes in all WGCNA modules from this manuscript, along with Spearman's correlations with two-sided significance of module genes with modules PC1 eigengene vectors. In addition, the genes in the four modules of interest from this paper are also shown in separate tabs for easier viewing (i.e. *saddlebrown*, *orange*, *darkgrey*, and *mediumpurple3*).

Supplementary Data 4 – In an effort to further investigate the importance of cognitive status in our samples, we ran 1000 iterations where half of the samples with pathology and reported cognitive impairment are being replaced with samples with pathology and no reported cognitive impairment (i.e. the blue analysis in Figure 3, panels A and B is having half of its pathology samples replaced with pathology samples from the green analysis). As shown in Supplementary Data 4, this did not statistically change the overall burden of pathology in any of the simulations using the two-sided Mann-Whitney U test. In contrast, all four of our modules fail to clear 0.1 FDR significance using the Benjamini-Hochberg procedure in their Spearman's correlation with β-amyloid and tau for the majority of the simulations. Taken together, these findings indicate that the correlations of these modules with AD pathology are highly sensitive to cognitive status.

Supplementary Data 5 - The eigengene of the *saddlebrown*, *orange*, *darkgrey*, and *mediumpurple3* modules was correlated with eight cell-type specific signatures from the human literature [1] (Inhibitory neurons, Excitatory neurons, Oligodendrocyte precursor cells (OPCs), Oligodendrocytes, Astrocytes, Microglia, Endothelial cells and Pericytes). Also shown are Spearman's correlations of these cell-type specific signatures with the mean gene expression vector of the subset of genes from each module that positively correlate with the module eigengene and the subset of genes that negatively correlate with the module eigengene. In addition, the enrichment for each module for these cell-type specific gene lists using the Fisher's exact test is shown, as are separate enrichment scores for module genes that positively and negatively correlate with the module eigengene. For both correlations and enrichment statistics, two-sided p-values and Bonferroni adjusted p-values are shown.

Supplementary Data 6 – Shown is the full ontology analysis for *saddlebrown*, *orange*, *darkgrey*, and *mediumpurple3* modules. In addition are shown the ontology analysis for genes that positively and negatively correlate with the PC1 eigengene for these four modules. Three categories of ontology analysis were investigated (molecular function(m), biological process (b) and cellular component(c)), with all categories included that have at least two shared genes with the test module. One-sided Fisher's exact test is calculated for each gene group. The q values were FDR adjusted across all the selected ontology terms within each category(m, b or c); see Methods for details.

Supplementary Data 7 – This table shows two-sided Fisher's exact test (FET) overlap between NPH modules and mouse microglial gene lists [2, 3] and publicly available human single-nuclei [1, 4, 5] and single-cell microglial gene lists [6, 7, 8, 9]. See main text for description of all studies shown in this table. For the three single-nuclei RNA-seq studies (Mathys et al. (2019) [1], Zhou et al. [4], and Grubman et al. [5]), differential expression of microglial genes is shown, and in these cases the total number of significant genes that pass FDR < 0.1 followed by positive and negative associations with AD are shown in parentheses (i.e. total(positive/negative)).

Supplementary Data 8 – Spearman's correlations, two-sided p-values, and Bonferroni adjusted p-values for panels A and B in Figure 5 are shown.

Supplementary Data 9 - Spearman's correlation of the *saddlebrown*, *orange*, *darkgrey*, and *mediumpurple3* module with all of the modules from Mostafavi et al. [10], as well as overlap (enrichment) using the Fisher's exact test. For both correlations and enrichment statistics, two-sided p-values and Bonferroni adjusted p-values are shown.

Supplementary Data 10 – Fisher's exact test overlap, two-sided p-values, and Bonferroni adjusted p-values for panels E-N in Figure 5 are shown.

Supplementary Data 11 – Spearman's correlations, two-sided p-values, and Bonferroni adjusted p-values for Supplementary Figure 4 are shown.

Supplementary Data 12 – Spearman's correlations, two-sided p-values, and Bonferroni adjusted p-values for Figure 8 are shown.

Supplementary Data 13 – Shown are the gene members from the top 5 microglial modules from our WGCNA analysis, along with Spearman's correlations with module PC1 eigengenes and membership in microglial gene lists from Keren-Shaul, et al. [2] and Mathys, et al. (2017) [3] (0 = not a member, 1 = is a member).

Source Data File – This data file contains the data points plotted in Supplementary Figure 2 and Supplementary Figure 5.

References:

1. Mathys H*, et al.* Single-cell transcriptomic analysis of Alzheimer's disease. *Nature* **570**, 332-337 (2019).

2. Keren-Shaul H*, et al.* A Unique Microglia Type Associated with Restricting Development of Alzheimer's Disease. *Cell* **169**, 1276-1290 e1217 (2017).

3. Mathys H*, et al.* Temporal Tracking of Microglia Activation in Neurodegeneration at Single-Cell Resolution. *Cell reports* **21**, 366-380 (2017).

4. Zhou Y*, et al.* Human and mouse single-nucleus transcriptomics reveal TREM2-dependent and TREM2-independent cellular responses in Alzheimer's disease. *Nature medicine* **26**, 131-142 (2020).

5. Grubman A*, et al.* A single-cell atlas of entorhinal cortex from individuals with Alzheimer's disease reveals cell-type-specific gene expression regulation. *Nature neuroscience* **22**, 2087-2097 (2019).

6. Hasselmann J*, et al.* Development of a Chimeric Model to Study and Manipulate Human Microglia In Vivo. *Neuron* **103**, 1016-1033 e1010 (2019).

7. Mancuso R*, et al.* Stem-cell-derived human microglia transplanted in mouse brain to study human disease. *Nature neuroscience* **22**, 2111-2116 (2019).

8. Olah M*, et al.* Single cell RNA sequencing of human microglia uncovers a subset associated with Alzheimer's disease. *Nat Commun* **11**, 6129 (2020).

9. Alsema AM*, et al.* Profiling Microglia From Alzheimer's Disease Donors and Non-demented Elderly in Acute Human Postmortem Cortical Tissue. *Front Mol Neurosci* **13**, 134 (2020).

10. Mostafavi S*, et al.* A molecular network of the aging human brain provides insights into the pathology and cognitive decline of Alzheimer's disease. *Nature neuroscience* **21**, 811-819 (2018).
