# Supplementary material for: An immune response characterizes early Alzheimer’s disease pathology and subjective cognitive impairment in hydrocephalus biopsies
Source: Nat Commun. 2021 Sep 27;12:5659. doi: 10.1038/s41467-021-25902-y (PMC8476497; doi:10.1038/s41467-021-25902-y)
Supplement: Supplementary file 2 — Description of Additional Supplementary Files [file 41467_2021_25902_MOESM2_ESM.docx]

Description of Additional Supplementary Files

Title: Supplementary Data 1

Description: List of all genes that correlate with β-amyloid and tau in the full dataset, also correlations with frontal samples only and parietal samples only, along with p-values and adjusted p-values using the Benjamini-Hochberg procedure. For the top 100 genes that correlate with β-amyloid in the full dataset, the Spearman’s correlation coefficient of these genes with β-amyloid in frontal and parietal samples are themselves highly correlated, with a Spearman’s correlation of r=0.8691309, and two-sided p-value<2.2E-16 across all 100 genes. Both frontal and parietal samples are equally similar to the full dataset as well, with a Spearman’s correlation of r = 0.9893544, and two-sided p-value<2.2E-16 when the correlation of these 100 genes with β-amyloid are compared between the full dataset vs. frontal samples, and r=0.9297355, and two-sided p-value<2.2E-16 when the comparison is between the full dataset and parietal samples. A similar relationship holds with tau; for the top 100 genes that correlate with tau in the full dataset, the correlation coefficient of these genes with tau in frontal and parietal samples have a Spearman’s correlation of r=0.7948915, and two-sided p-value <2.2E-16. When the correlation of these 100 genes with tau are compared between the full dataset vs. frontal samples, r = 0.9769622, and two-sided p-value<2.2E-16, and when the correlation of these 100 genes with tau are compared between the full dataset vs. parietal samples, r=0.9037152, and two-sided p-value<2.2E-16. Also included in this table file are the p-values and adjusted p-values that correspond to a two-sided Fisher’s exact test enrichment for cell-type specific genes among the genes that individually past FDR 0.1 threshold in the full dataset, using human single-nucleus RNA-seq data 1. Finally, in order to further test the overall consistency of gene expression in these biopsies, we performed differential gene expression, and directly compared biopsy tissue with no AD pathology (no β-amyloid or tau, n = 32) to biopsy tissue with any AD pathology (either β-amyloid and/or tau, n = 74). This analysis only yielded two genes that passed FDR of 0.1. Similar analyses based only on β-amyloid (no β-amyloid, n = 49 vs. any β-amyloid, n = 57) or tau (no tau, n = 42 vs. any tau, n = 64) yielded 19 and 4 genes passing FDR 0.1 respectively. See main text for discussion and analysis.

Title: Supplementary Data 2

Description: Spearman’s correlation of all WGCNA modules with β-amyloid and tau pathology in all samples, in the remove CI-path group, and in the remove non-CI path group from Figure 3, along with two-sided p-values and adjusted p-values using the Benjamini-Hochberg procedure.

Title: Supplementary Data 3

Description: Shown are all genes in all WGCNA modules from this manuscript, along with Spearman’s correlations with two-sided significance of module genes with modules PC1 eigengene vectors. In addition, the genes in the four modules of interest from this paper are also shown in separate tabs for easier viewing (i.e. saddlebrown, orange, darkgrey, and mediumpurple3).

Title: Supplementary Data 4

Description: In an effort to further investigate the importance of cognitive status in our samples, we ran 1000 iterations where half of the samples with pathology and reported cognitive impairment are being replaced with samples with pathology and no reported cognitive impairment (i.e. the blue analysis in Figure 3, panels A and B is having half of its pathology samples replaced with pathology samples from the green analysis). As shown in Supplemental Data 4, this did not statistically change the overall burden of pathology in any of the simulations using the two-sided Mann-Whitney U test. In contrast, all four of our modules fail to clear 0.1 FDR significance using the Benjamini-Hochberg procedure in their Spearman’s correlation with β-amyloid and tau for the majority of the simulations. Taken together, these findings indicate that the correlations of these modules with AD pathology are highly sensitive to cognitive status.

Title: Supplementary Data 5

Description: The eigengene of the saddlebrown, orange, darkgrey, and mediumpurple3 modules was correlated with eight cell-type specific signatures from the human literature 1 (Inhibitory neurons, Excitatory neurons, Oligodendrocyte precursor cells (OPCs), Oligodendrocytes, Astrocytes, Microglia, Endothelial cells and Pericytes). Also shown are Spearman’s correlations of these cell-type specific signatures with the mean gene expression vector of the subset of genes from each module that positively correlate with the module eigengene and the subset of genes that negatively correlate with the module eigengene. In addition, the enrichment for each module for these cell-type specific gene lists using the Fisher's exact test is shown, as are separate enrichment scores for module genes that positively and negatively correlate with the module eigengene. For both correlations and enrichment statistics, two-sided p-values and Bonferroni adjusted p-values are shown.

Title: Supplementary Data 6

Description: Shown is the full ontology analysis for saddlebrown, orange, darkgrey, and mediumpurple3 modules. In addition are shown the ontology analysis for genes that positively and negatively correlate with the PC1 eigengene for these four modules. Three categories of ontology analysis were investigated (molecular function(m), biological process (b) and cellular component(c)), with all categories included that have at least two shared genes with the test module. One-sided Fisher’s exact test is calculated for each gene group. The q values were FDR adjusted across all the selected ontology terms within each category(m, b or c); see Methods for details.

Title: Supplementary Data 7

Description: This table shows two-sided Fisher’s exact test (FET) overlap between NPH modules and mouse microglial gene lists 2, 3 and publicly available human single-nuclei 1, 4, 5 and single-cell microglial gene lists 6, 7, 8, 9. See main text for description of all studies shown in this table. For the three single-nuclei RNA-seq studies (Mathys et al. (2019) 1, Zhou et al. 4, and Grubman et al. 5), differential expression of microglial genes is shown, and in these cases the total number of significant genes that pass FDR < 0.1 followed by positive and negative associations with AD are shown in parentheses (i.e. total(positive/negative)).

Title: Supplementary Data 8

Description: Spearman’s correlations, two-sided p-values, and Bonferroni adjusted p-values for panels A and B in Figure 5 are shown.

Title: Supplementary Data 9

Description: Spearman’s correlation of the saddlebrown, orange, darkgrey, and mediumpurple3 module with all of the modules from Mostafavi et al. 10, as well as overlap (enrichment) using the Fisher's exact test. For both correlations and enrichment statistics, two-sided p-values and Bonferroni adjusted p-values are shown.

Title: Supplementary Data 10

Description: Fisher's exact test overlap, two-sided p-values, and Bonferroni adjusted p-values for panels E-N in Figure 5 are shown.

Title: Supplementary Data 11

Description: Spearman’s correlations, two-sided p-values, and Bonferroni adjusted p-values for Supplemental Figure 4 are shown.

Title: Supplementary Data 12

Description: Spearman’s correlations, two-sided p-values, and Bonferroni adjusted p-values for Figure 8 are shown.

Title: Supplementary Data 13

Description: Shown are the gene members from the top 5 microglial modules from our WGCNA analysis, along with Spearman’s correlations with module PC1 eigengenes and membership in microglial gene lists from Keren-Shaul, et al. 2 and Mathys, et al. (2017) 3 (0 = not a member, 1 = is a member).
