# Supplementary material for: An immune response characterizes early Alzheimer’s disease pathology and subjective cognitive impairment in hydrocephalus biopsies
Source: Nat Commun. 2021 Sep 27;12:5659. doi: 10.1038/s41467-021-25902-y (PMC8476497; doi:10.1038/s41467-021-25902-y)
Supplement: Supplementary file 16 — Reporting Summary [file 41467_2021_25902_MOESM16_ESM.pdf]

nature research

Corresponding author(s): Andrew F Teich

Last updated by author(s): Jun 22, 2021

Reporting Summary

Nature Research wishes to improve the reproducibility of the work that we publish. This form provides structure for consistency and transparency in reporting. For further information on Nature Research policies, see our Editorial Policies and the Editorial Policy Checklist.

Statistics

For all statistical analyses, confirm that the following items are present in the figure legend, table legend, main text, or Methods section.

n/a	Confirmed	
☐	☒	The exact sample size (*n*) for each experimental group/condition, given as a discrete number and unit of measurement
☐	☒	A statement on whether measurements were taken from distinct samples or whether the same sample was measured repeatedly
☐	☒	The statistical test(s) used AND whether they are one- or two-sided<br>*Only common tests should be described solely by name; describe more complex techniques in the Methods section.*
☐	☒	A description of all covariates tested
☐	☒	A description of any assumptions or corrections, such as tests of normality and adjustment for multiple comparisons
☐	☒	A full description of the statistical parameters including central tendency (e.g. means) or other basic estimates (e.g. regression coefficient) AND variation (e.g. standard deviation) or associated estimates of uncertainty (e.g. confidence intervals)
☐	☒	For null hypothesis testing, the test statistic (e.g. *F*, *t*, *r*) with confidence intervals, effect sizes, degrees of freedom and *P* value noted<br>*Give P values as exact values whenever suitable.*
☒	☐	For Bayesian analysis, information on the choice of priors and Markov chain Monte Carlo settings
☒	☐	For hierarchical and complex designs, identification of the appropriate level for tests and full reporting of outcomes
☒	☐	Estimates of effect sizes (e.g. Cohen's *d*, Pearson's *r*), indicating how they were calculated

Our web collection on statistics for biologists contains articles on many of the points above.

Software and code

Policy information about availability of computer code

Data collection	No software was used for data collection
Data analysis	All analysis software used in this manuscript is publicly available and is described in detail in our methods section. These analysis pipelines include: FastQC v 0.11.8, STAR 2.6.0a, ConsensusPathDB 34, featureCounts 1.5.0-p1, DESeq2 1.10.1, SVA 3.18.0, limma 3.26.9, WGCNA 1.66, biomaRt 2.37.6, and cor.test and p.adjust from R 3.2.3

For manuscripts utilizing custom algorithms or software that are central to the research but not yet described in published literature, software must be made available to editors and reviewers. We strongly encourage code deposition in a community repository (e.g. GitHub). See the Nature Research guidelines for submitting code & software for further information.

Data

Policy information about availability of data

All manuscripts must include a data availability statement. This statement should provide the following information, where applicable:

- Accession codes, unique identifiers, or web links for publicly available datasets
- A list of figures that have associated raw data
- A description of any restrictions on data availability

The RNA-seq data generated in this study have been deposited in the synapse.org (Sage Bionetworks) database under DOI: 10.7303/syn21898410 [https://www.synapse.org/#!Synapse:syn21898410/wiki/603968]. The RNA-seq data and associated metadata are available under restricted access due to the fact that this data is derived from human tissue samples, and access can be obtained by making a formal request through the synapse.org database.

Field-specific reporting

Please select the one below that is the best fit for your research. If you are not sure, read the appropriate sections before making your selection.

[x] Life sciences [ ] Behavioural & social sciences [ ] Ecological, evolutionary & environmental sciences

For a reference copy of the document with all sections, see nature.com/documents/nr-reporting-summary-flat.pdf

Life sciences study design

All studies must disclose on these points even when the disclosure is negative.

Sample size	In this study, we perform RNA-seq on 106 NPH samples that were banked at our institution under IRB-AAAA4666, which allows for the distribution of de-identified tissue samples and clinical data to researchers as "Not Human Subject Research." We sequenced all available NPH samples that were banked under this protocol at the time the study was done that had a RIN of 6 or higher.
Data exclusions	As stated in our manuscript, we sequenced all samples with a RIN score of 6 and higher; no other exclusion criteria was used; this led to sequencing of 106 samples.
Replication	In our manuscript, we show that our findings translate to established autopsy-based AD RNA-seq datasets. As we show in our manuscript, three of our WGCNA modules that correlate with AD pathology in our NPH data (saddlebrown, orange, and darkgrey) also correlate with AD pathology in bulk RNA-seq date from AD autopsy tissue, while our mediumpurple3 module does not. This prompts an extensive comparison with other autopsy datasets that support the notion that a decline in homeostatic genes in AD that is seen in our NPH data is generally not seen in other autopsy datasets, with the exception of entorhinal cortex data.
Randomization	This is a retrospective study, and so there is no randomization of subjects because we did not experiment on our subjects. As stated in our Methods, we regressed out variability in gene expression not attributable to our primary variables of interest (beta-amyloid and tau) using surrogate variable analysis, which is a commonly used method to identify known and unknown confounders and covariates in data.
Blinding	All biopsy samples were processed and sequenced with researchers blinded to any associated metadata. Analysis of immunofluorescent images (Figure 8) was performed blinded to patient's cognitive status.

Reporting for specific materials, systems and methods

We require information from authors about some types of materials, experimental systems and methods used in many studies. Here, indicate whether each material, system or method listed is relevant to your study. If you are not sure if a list item applies to your research, read the appropriate section before selecting a response.

Materials & experimental systems

n/a	Involved in the study	
[ ]	[x]	Antibodies
[x]	[ ]	Eukaryotic cell lines
[x]	[ ]	Palaeontology and archaeology
[x]	[ ]	Animals and other organisms
[ ]	[x]	Human research participants
[x]	[ ]	Clinical data
[x]	[ ]	Dual use research of concern

Methods

n/a	Involved in the study	
[x]	[ ]	ChIP-seq
[x]	[ ]	Flow cytometry
[x]	[ ]	MRI-based neuroimaging

Antibodies

Antibodies used	As stated in the manuscript, immunohistochemical data from the clinical biopsies from these patients was used in this manuscript; this data was generated using the Columbia University Department of Pathology clinical service. The antibodies this service uses for these biopsies are tau (AT8 at 1:200 dilution; Thermo Fisher; Catalog # MN1020), beta-amyloid (6E10 at 1:200 dilution; BioLegend; Catalog # 803003), alpha-synuclein (KM51 at 1:40 dilution; Leica; Catalog # NCL-L-ASYN), and TDP-43 (C-terminal rabbit polyclonal at 1:500 dilution; Proteintech; Catalog # 12892-1-AP). We also conducted our own staining on tissue sections from these patients, and we used a beta-amyloid antibody to visualize amyloid plaques (Cell signaling at 1:200 dilution; Catalog # 15126, lot 1) and an IBA-1 antibody to visualize microglia (Wako Catalog # 019-19741 at 1:500 dilution, lot ptr2404), along with Alexa Fluor 555 conjugated Donkey Anti-Mouse (Invitrogen A-31570 at 1:1000 dilution, lot 1850121) and Alexa Fluor 488 conjugated Donkey Anti-Rabbit (Invitrogen A-21206 at 1:1000 dilution, lot 1981155).
Validation	The antibodies that the hospital uses in pathologic diagnosis (the first four mentioned above) have their quality continuously monitored on positive and negative control tissue by the clinical service, as these antibodies are used by the Pathology Department in the diagnosis of clinical samples. The antibodies that we used ourselves (the last two mentioned above) have been previously validated for IF and IHC by the manufacturers, as shown on their websites. More importantly, the beta-amyloid antibody stains plaques in NPH biopsy tissue that reportedly had beta-amyloid plaques on clinical examination. The IBA-1 antibody also stains

microglial-appearing cells in the NPH biopes. In addition, the IBA-1 antibody we used is one of the most commonly used antibodies for visualizing microglia, and the manufacturer has 14 pages of citations, including 18 Nature papers and 14 Cell papers. More detailed validation data is below:

Anti-Phospho-Tau (MN1020): Western blot data showing the specificity of antibody binding to phospho-tau can be found on the manufacturer website at https://www.thermofisher.com/antibody/product/Phospho-Tau-Ser202-Thr205-Antibody-clone-AT8-Monoclonal/MN1020 ; used to identify neurofibrillary tangles in human brain immunohistochemistry cited in PMID 26936765.
Anti-β-Amyloid (6E10): Western blot data showing specificity for human β-Amyloid with no binding to rodent Aβ1-42, and human brain immunohistochemistry data staining plaques found on the manufacturer website https://www.biolegend.com/en-us/products/purified-anti-beta-amyloid-1-16-antibody-11228.
Anti-α-Synuclein (KM51): KM51 displayed no non-specific staining of the neuropil (PMID: 16640654) ; human brain immunohistochemistry staining of Lewy bodies found on the manufacturer website https://shop.leicabiosystems.com/us/ihc-ish/ihc-primary-antibodies/pid-alpha-synuclein.
Anti-TDP-43 (Proteintech # 12892-1-AP): knock-down validation in A549 cells found on the manufacturer website https://www.ptglab.com/products/TARDBP-Antibody-12892-1-AP.htm
Anti-β-Amyloid (Cell Signaling # 15126): Western blot data displaying binding with human Aβ-37, Aβ-40, and Aβ-42 and application in human brain immunohistochemistry staining plaques can be found on the manufacturer website: https://www.cellsignal.com/products/primary-antibodies/b-amyloid-d3d2n-mouse-mab/15126
IBA-1 (Wako # 019-19741): Immunohistochemical staining of microglia found on the manufacturer website https://labchem-wako.fujifilm.com/us/product/detail/W01W0101-1974.html; Immunohistochemistry in human white matter co-expressing IBA-1 and TMEM-119 on microglia cited at PMID: 29804289.
Alexa Fluor 555 conjugated Donkey Anti-Mouse (Invitrogen A-31570): From the manufacturer website (https://www.thermofisher.com/us/en/home/life-science/antibodies/antibody-performance-guarantee.html): "To minimize cross-reactivity, these donkey anti-mouse IgG whole antibodies have been affinity-purified and show minimum cross-reactivity to bovine, chicken, goat, guinea pig, hamster, horse, human, rabbit, rat, and sheep serum proteins. Cross-adsorption or pre-adsorption is a purification step to increase specificity of the antibody resulting in higher sensitivity and less background staining." Application in human brain immunohistochemistry cited in PMID: 28165465.
Alexa Fluor 488 conjugated Donkey Anti-Rabbit (Invitrogen A-21206): From the manufacturer website (https://www.thermofisher.com/us/en/home/life-science/antibodies/antibody-performance-guarantee.html): "To minimize cross-reactivity, these donkey anti-rabbit IgG whole antibodies have been affinity-purified and show minimum cross-reactivity to bovine, chicken, goat, guinea pig, hamster, horse, human, mouse, rat, and sheep serum proteins. Cross-adsorption or pre-adsorption is a purification step to increase specificity of the antibody resulting in higher sensitivity and less background staining." Application in human brain immunohistochemistry cited in PMID: 30202002.

Human research participants

Policy information about studies involving human research participants

Population characteristics	The covariate relevant population characteristics include age, gender, RIN (RNA integrity number) of the biopsy material, as well as sequencing batch of the biopsies.
Recruitment	This study is a retrospective study that uses residual tissue samples not required for clinical diagnosis and associated clinical and demographic data. At the time of surgery, a portion of tissue was frozen and deposited in a tissue bank repository by a tissue bank staff member, and a portion of tissue was submitted for clinical workup. Tissue and clinical data stripped of patient identifiers was released to the research team by the tissue bank. Workup of clinically submitted biopsy material was complete at the time of residual tissue release by the tissue bank. This study was reviewed and approved by the Columbia University Institutional Review Board (IRB), and all relevant ethical regulations have been followed.
Ethics oversight	This study was reviewed and approved by the Columbia University Institutional Review Board (IRB), and all relevant ethical regulations have been followed.

Note that full information on the approval of the study protocol must also be provided in the manuscript.
